# Carbon–phosphorus exchange rate constrains density–speed trade-off in arbuscular mycorrhizal fungal growth

**DOI:** 10.1073/pnas.2512182123

**Published:** 2026-02-06

**Authors:** Corentin Bisot, Loreto Oyarte Galvez, Félix Kahane, Marije van Son, Bianca Turcu, Rob Broekman, Kai-Kai Lin, Paco Bontenbal, Max Kerr Winter, Vasilis Kokkoris, Stuart A. West, Christophe Godin, E. Toby Kiers, Thomas S. Shimizu

**Affiliations:** ^a^Amsterdam Institute for Life and Environment, Faculty of Science, Section Systems Ecology, Vrije Universiteit Amsterdam, Amsterdam 1081 HV, The Netherlands; ^b^Infomatter Departments, AMOLF, Amsterdam 1098 XG, The Netherlands; ^c^Laboratoire de Reproduction et Développement des Plantes, Université de Lyon, Ecole Normale Supérieure de Lyon, Université Claude Bernard Lyon 1, Institut national de recherche pour l’agriculture, l’alimentation et l’environnement (INRAE), CNRS, 69364 Lyon Cedex 07, France; ^d^Department of Biology, University of Oxford, Oxford OX1 3SZ, United Kingdom; ^e^Society for the Protection of Underground Networks, Dover, DE 19901

**Keywords:** network, symbiosis, fungi

## Abstract

Symbiotic nutrient exchange between arbuscular mycorrhizal (AM) fungi and their host plants varies widely depending on their physical, chemical, and biological environment. Yet dissecting this context dependency remains challenging because we lack methods for tracking nutrients such as carbon (C) and phosphorus (P). Here, we developed an approach to quantitatively estimate C and P fluxes in the AM symbiosis from comprehensive network morphology quantification, achieved by robotic imaging and machine learning based on roughly 100 million hyphal shape measurements. We found that rates of C transfer from the plant and P transfer from the fungus were, on average, related proportionally to one another. This ratio was nearly invariant across AM fungal strains despite contrasting growth phenotypes but was strongly affected by plant host genotype. Fungal phenotype distributions were bounded by a Pareto front with a shape favoring specialization in an exploration–exploitation trade-off. This means AM fungi can be fast range expanders or fast resource extractors, but not both. Manipulating the C/P exchange rate by swapping the plant host genotype shifted this Pareto front, indicating that the exchange rate constrains possible AM fungal growth strategies. We show by mathematical modeling how AM fungal growth at fixed exchange rate leads to qualitatively different symbiotic outcomes depending on fungal traits and nutrient availability.

## Significance

The exchange of phosphorus and carbon between arbuscular mycorrhizal fungi and plants is fundamental to both global ecosystem productivity and the regulation of the Earth’s climate. In this paper, we analyzed hundreds of timelapses of fungal network growth using an automated pipeline. This allowed us to simultaneously track the time course of resource exchange during symbiotic growth. This approach provides a basis for understanding and predicting the context dependence of nutrient exchange, as well as the control mechanisms governing a symbiosis evolved over 400 My.

The symbiotic partnership between arbuscular mycorrhizal (AM) fungi and their host plants is one of the most ubiquitous symbioses on Earth, with 70% of all plant species forming these resource exchange partnerships. Plants provide AM fungi with carbon in the form of sugars and fats, while phosphorus (P) and other nutrients are provided by fungal partners. In some cases, fungi have been shown to provide up to 80% of a host plant’s P supply (1). In return, plants deliver on average 6% of their photosynthetically fixed carbon (C) to AM fungal partners (2). This partnership helped facilitate the colonization of land by plants over 400 Mya, and today it is fundamental to both global ecosystem productivity and the regulation of the Earth’s climate (1).

While the importance of nutrient trade between AM fungi and plants is well recognized, resource exchange is highly context dependent. Biotic and abiotic conditions influence the trade of nutrients in ways that we do not fully understand. This context dependency makes it difficult to predict cumulative nutritional effects of the symbiosis, such as the total amount of phosphorus transferred by the fungal partner to its host over a given time. Specifically, research has shown that fungal strains differ in the amounts of phosphorus that they supply to their host plants (3–5). These differences have been assumed to be related to differences in fungal traits and/or fungal trading strategies over space and time (6, 7).

We anticipate that fungal strategies are under selection for multiple, potentially conflicting tasks. Limited resources can be invested in exploration, local densification, reproduction, or feeding the hyphal surface microbiome (8). Such multiobjective optimizations under constraints often give rise to performance trade-offs identifiable in the form of Pareto fronts, boundaries in trait space that delineate achievable fungal phenotypes under constraints such as resource limitation (9). Resolving such Pareto fronts from experimental data can be informative of the type of constraints that underlie complex phenotypes such as morphogenesis.

Indeed, plants appear to adjust the amount of carbon they supply to fungi in response to how much phosphorus they are supplied, a principle often referred to as reciprocal exchange (10–12).

However, the mechanisms that underlie such changes in nutrient exchange, and the strategies they might represent remain unclear (13, 14). Do plants and fungi have fixed trading rules, or do they modulate their trade behaviors depending on nutrient availability and/or the specific partners they interact with? And within a given plant-fungal pair, is the relative amount of nutrients received by each partner (i.e. the effective exchange rate) fixed in a manner that guarantees steady returns, or does it vary in a manner that might reflect more flexible strategies at play (e.g. cooperation vs. cheating)? Past work has quantified the C/P-exchange ratio in relative terms using isotope labeling (11, 15). Yet, such end-point measurements are laborious and are often noisy due to the multiple extraction steps involved. Furthermore, they yield only a single ratio value per mycorrhizal replicate. An ideal experiment would allow tracking of both carbon and phosphorus transfer over time within each replicate. However, so far such dynamical in vivo tracking of two-way resource transfer has remained out of reach.

Here, we addressed this challenge by developing an approach based on high-throughput robotic imaging and machine learning that enables estimation of both carbon expenditure by the plant and phosphorus delivery by the fungus. Because the imaging measurements are noninvasive, we are able to obtain these estimates at regular (typically 2-h) intervals as the mycorrhizal network develops. The key idea is that both the carbon cost of network growth (which dominates fungal carbon expenditure) and phosphorus uptake rate (which limits phosphorus transfer to the plant) can be estimated from precise measurements of hyphal morphology if such measurements can be conducted comprehensively throughout the entire network and during steady-state growth. The basic rationale is that both building costs and nutrient uptake rates of the hyphal network scale with morphological observables. First, the amount of carbon required for network construction is to a reasonable approximation proportional to the total volume V of the hyphal network (16). Second, the ability of the network to absorb solutes such as phosphorus from the environment and transfer them to the plant host is expected to depend on its total membrane surface area, S. We were able to quantify this dependence with direct measurements of phosphorus depletion and translocation to calibrate the dependence of phosphorus absorption and transfer to the host root on the network surface area S. Thus, sufficiently accurate measurements of morphology across the entire network, with appropriate calibrations, can enable reliable quantification of transferred carbon and phosphorus.

**Fig. 1.**
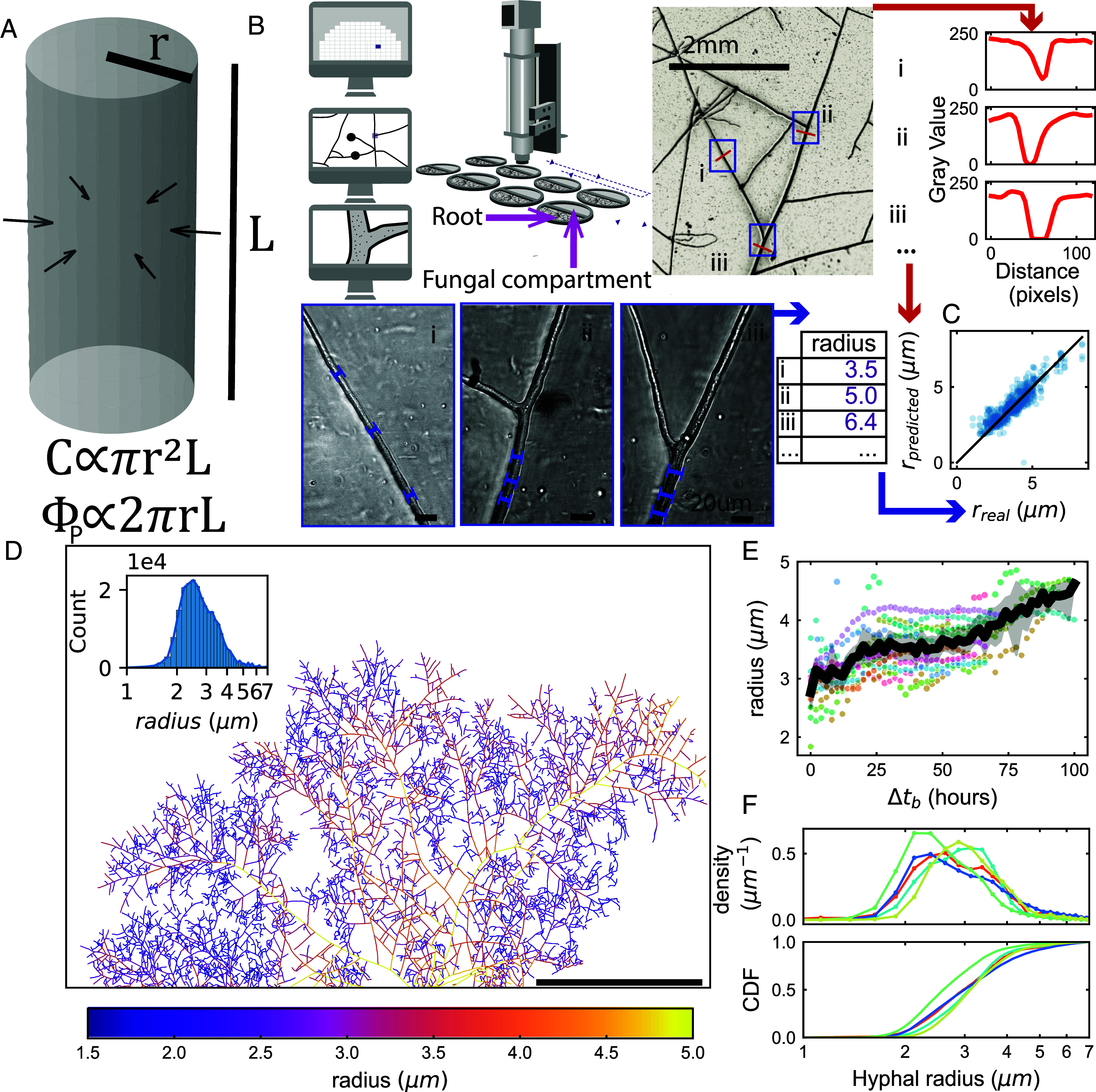
Hyphal radius variation in space, time, and across genotypes as revealed by a CNN machine learning model. (*A*) Illustration showing the importance of hyphal radius for fungal resource economy. Cost of building a hyphal segment of length L and radius r is proportional to cylinder volume while absorption of solute from the environment is proportional to cylinder surface area. (*B*) Description of the dataset generation and training scheme. Images are acquired in low (scale bar is 2 mm) and high magnification (scale bar is 20 μm) at different location in the network. Hyphal radius is manually measured on the high magnification images and orthogonal 120 px transect are extracted from the low magnification images. A Convolutional Neural Network (CNN) is trained to predict the measured radius from the extracted transect. (*C*) Evaluation of the trained neural network on an independent dataset that has not been used for training (RMSE = 0.70, R2 = 0.66). The black line is the identity function, blue points are individual predictions from the independent dataset. (*D*) Network graph where each edge color is mapped to the radius value estimated with the trained CNN. *Inset* is the distribution of radius value over all 10px segments that constitute the graph. (*E*) Temporal variation in radius of a selection of (n=13) major hyphae of a network as a function of the time since their emergence after branching. Each dot color correspond to a given hypha. The black line corresponds to average and gray shade to 95% CI computed on all points over intervals of two hours. (*F*) *Upper* Volume weighted distribution of the radius of all edges at final timestep for different fungal strains and species: *R. irregularis* A5 (cyan, n=8), C2 (blue, n=19) and C3 (red, n=4), *R. aggregatus* (green, n=4), and *R. clarus* (yellow, n=4). Curves are linking the top of histogram bins. *Lower* Cumulative distribution function representing for each radius the proportion of total network volume that is contributed by hyphal element below that radius.

To precisely and comprehensively measure hyphal morphology across the entire network, we leveraged a robotic imaging and analysis pipeline we recently developed (17), capable of extracting the full network graph of the extraradical mycelium (ERM) of AM fungi over time, after forming symbiosis with an in vitro root-organ-cultures (ROC) as host. In each of these samples, the colonized host was restricted to the root compartment, while the fungal network crossed a physical barrier to a second compartment (i.e. fungal side) lined with a permeable cellophane layer to optimize visualization and constrain fungal network growth to the two-dimensional plane above the hydrogel medium (Fig. 1). Given the approximately cylindrical geometry of hyphal filaments (Fig. 1*A*), the relevant morphological parameters are the hyphal radius r and total hyphal length L, which are related to surface area and volume by simple scaling relationships (S∼rL and $V∼r^{2}L$). While the machine vision techniques developed in ref. 17 sufficed to yield accurate measurements of the network length L, challenges remained in accurate determination of the hyphal radius r, given the relatively low magnification at which the robot images the hyphal network. Past estimates of AM hyphal radii vary over a broad range, from 0.6 to 9 μm (a 15-fold difference) (18), but whether that diversity reflects changes over time, variation across space, and/or differences between strains/species remained largely unknown. We therefore developed a machine-learning-based method for precisely extracting r for every hyphal edge within growing extraradical mycorrhizal (ERM) networks. This method allowed us to obtain roughly one million hyphal radius measurements per network timelapse, and we applied it to ∼100 timelapses in this study.

For the validity of our morphology-based approach to estimating C/P exchange, it is important that measurements are made during steady-state growth, in which the cell’s elemental composition (19), as well as gene expression (20, 21) is constant. We ensured that the AM fungal network was in the steady-state growth regime by restricting our measurements to early times (<200 h) after hyphal crossing into the fungal compartment. This is when the ERM network expands as a traveling-wave, with constant speed and density (17). Whereas carbon cost will be to a good approximation proportional to the network volume V under these conditions, the dependence of phosphorus transfer on surface area S requires some model of phosphorus uptake/transport. We therefore conducted direct measurements of phosphorus depletion and translocation to validate and calibrate a minimal model that captures the dependence of phosphorus transport on S.

Combined with this calibrated model, robotic imaging and analysis of network morphology allowed us to dynamically track how AM fungi utilize plant-derived carbon to acquire and transfer phosphorus to the plant. We quantified these dynamics for multiple combinations of plant × fungus × phosphorus concentration to investigate the statistics of C/P exchange across time and how this varied with changes in trade partners and environmental conditions. Remarkably, we found that despite considerable variation in C/P exchange across samples and across time, on average, the amount of phosphorus transferred to the plant was approximately proportional to the amount of received carbon. Finally, we analyzed the significance of this proportional exchange for symbiotic trade using the framework of Pareto fronts, and extending a model of traveling-wave growth of AM fungal networks to account for C/P exchange.

## Machine-Learning Model Reveals Temporal, Spatial, and Genetic Variation in Hyphal Width

Because both carbon expenditure and phosphorus absorbing capacity of AM fungal networks depend on the width of hyphae across the network, we began by measuring hyphal radii from three different AM fungal strains over a 5 to 6 d period. To obtain network-wide hyphal morphology measurements, we developed a machine-learning-based approach to extract hyphal widths from data generated by our high-throughput imaging robot (Fig. 1*B*).

We trained a convolutional neural network (CNN) to extract the radius r of hyphal filaments from one-dimensional intensity linescans of hyphal transects within low-magnification output images of the robot (Fig. 1*B*). We selected approximately 1,000 positions from 6 fungal networks and imaged them at both low (2×) and high (50×) magnification. To generate ground truth data for training, we first manually measured the hyphal radius r within all high-resolution images. Subsequently, transect linescans orthogonal to the hyphal axis at those same positions were automatically extracted from corresponding low-magnification images. We then used the ground truth data as labels and the linescan data as features to train a two-layer CNN to extract r. We evaluated the CNN’s performance on an independent test set (RMSE = 0.70, R2 = 0.66), confirming that the model could reliably predict r from data outside the training set (Fig. 1*C* and *SI Appendix*, Fig. S1).

The resulting model can be used to efficiently extract r for every hyphal edge of the fungal network (Fig. 1*D*). We found that the radius of hyphae r was distributed broadly (Fig. 1*D*, *Inset*), from around 1 μm for the thinnest hyphae of branched absorbing structures (BAS; key sites for nutrient uptake) to about 6 to 7 μm for the thickest runner hyphae (RH). In addition to this spatial variation, we observed that RH tended to increase their width over time, with values of r growing, on average, from 3 μm to 4.5 μm over 100 h (Fig. 1*E*). Such increases were not due solely to cell wall thickening, because the cell wall never contributed to more than 20% of total hyphal width (*Materials and Methods*). This hyphal widening therefore represents increases in both plasma membrane surface area and cell volume. We also ruled out that these temporal changes were artifacts of the CNN model by confirming hyphal widening in manually measured subsets of edges imaged over multiple days at high magnification (*SI Appendix*, Fig. S2).

Finally, we tested how the distribution of the hyphal radius r varied across AM fungal genotypes, focusing on *Rhizophagusirregularis* A5, C2, and C3, *Rhizophagus aggregatus*, and *Rhizophagus clarus* (*Materials and Methods*), using the CNN model to predict r for all network edges. The choice of genotypes was driven by the motivation to sample broadly across the variation in network growth phenotypes and to ensure sufficient coverage across the range of fungal traits across strains compatible with in vitro culture. These measurements revealed that i) the r-distribution was broad and overlapped considerably across all tested genotypes (Fig. 1*F*, *Upper*), and yet ii) there were also significant differences across genotypes, with some strains tending to have smaller or larger hyphal radii, as shown by the cumulative distribution (Fig. 1*F*, *Lower*).

## Pattern of Fungal Growth Suggests Progressive Relaxation of Carbon Constraint

To place these contrasting hyphal radius distributions in the context of carbon costs, we next quantified the spatial density of hyphal filaments ρ (μm/mm^2^), as well as the traveling-wave range expansion speed $v_{\text{wave}}$ of the network. For a given network edge i of length $L_{i}$ and radius $r_{i}$, we approximated its volume $V_{i}$ as a cylinder, $V_{i}=\pi r_{i}^{2}L_{i}$. Using methods developed in ref. 17, we also detected all spores of the network and extracted their radius. For a given spore k of radius $r_{k}$ we estimated its volume as a sphere, $V_{k}=\frac{4}{3}\pi r_{k}^{3}$. At later times, spores can account for as much as 10 to 20% of total fungal biovolume (17). The sum of all hyphal and spore volumes could in turn be used to compute the total amount of carbon in the network $C_{t}$

[1] $$\begin{array}{c} C_{t}=M_{C}\left(\sum_{i\in edges}V_{i}+\sum_{k\in spores}V_{k}\right), \end{array}$$

where $M_{C}$ is the mass of carbon per unit cell volume. $M_{C}$ is obtained with the formula as $M_{C}=d_{\mathrm{cell}}f_{\mathrm{dry}}f_{\mathrm{carbon}}$, where $d_{\mathrm{cell}}$ is the mass density of the hyphal cell, $f_{\mathrm{dry}}$ is the dry mass fraction of the total (wet) mass, $f_{\mathrm{carbon}}$ is the fraction of dry mass accounted for by carbon. We note that while $M_{C}$ can be expected to be constant during steady-state growth (16), its precise value for AM fungi remains unknown and hence needs to be approximated (*SI Appendix*, section 4.A). Using $C_{t}$ given by Eq. **1**, we define the spatial carbon density $\rho_{C}\equiv dC_{t}/dA$, where A is the area of the network, computed as its convex hull (17).

**Fig. 2.**
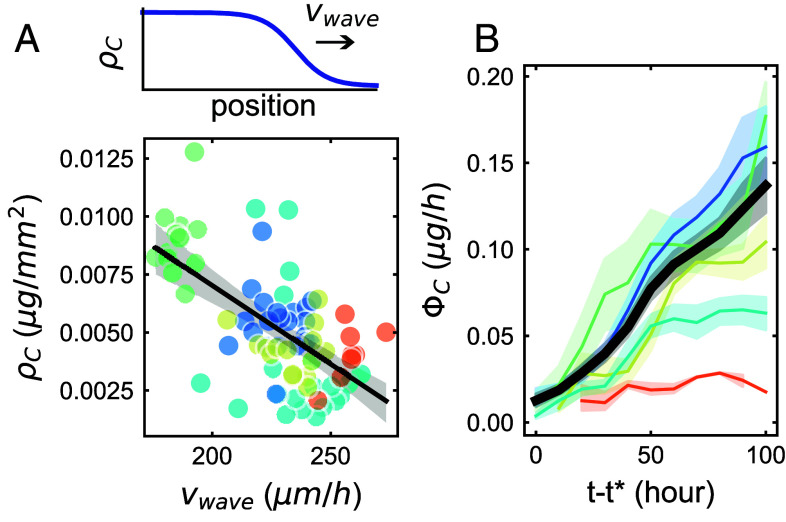
Carbon expenditure pattern of various fungal strains. (*A*) *Top*: Illustration of typical fungal density wave dynamic. *Bottom* Traveling-wave observables. $\rho_{C}$ is the instantaneous density of carbon across the network’s spatial range and $v_{\text{wave}}$ is the instantaneous wave speed. Each small circle corresponds to an independent median of those parameters over 10 h for all plates of the same strain (*n*= 7 to 11). Black line represent bootstrap regression on all datapoints. Gray shade correspond to 95% C.I. of the linear fit obtained via bootstrapping. (*B*) Carbon spent by the growing network in hyphal structures per hour as a function of time. The black line and shade represent average and 95% CI of all replicates’ dynamics. Colored lines correspond to average and 95% confidence over all replicates for one strain. Average and 95% CI (mean ±2× s.e.m.) are computed over 10-h time intervals. For all plots, color correspondence and number of replicates are the following: *R. irregularis* A5 (cyan, n=9), C2 (blue, n=19) and C3 (red, n=3), *R. aggregatus* (green, n=4), and *R. clarus* (yellow, n=4).

We found that fungal networks that expanded faster (i.e. with greater $v_{\text{wave}}$) tended to grow sparser (i.e. at lower $\rho_{C}$) and vice versa (Fig. 2*A*). To further investigate whether this negative correlation between speed and density was robust across different plant hosts, we measured $\rho_{C}$ and $v_{\text{wave}}$ for three of the fungal strains in combination with two different host genotypes. We chose two different genotypes of carrot *Daucus carota* as these hosts (hereafter referred to as genotype 1 and genotype 2) grow at different rates, with genotype 1 growing slowly and genotype 2 growing fast (17). We found that while both $v_{\text{wave}}$ and $\rho_{C}$ were affected by host genotype, they changed in a manner that preserved the negative correlation (*SI Appendix*, Fig. S3). Specifically, networks grown with genotype 2 carrot root expanded their spatial range faster than those grown with genotype 1 carrot root, but when attached to either host, *R. irregularis* A5 grew sparser and expanded faster than *R. irregularis* C2, itself faster and sparser than *R. aggregatus*.

Both range expansion speed (promoting exploration) and hyphal density (promoting exploitation) are phenotypic traits of network growth. The existence of a negative correlation between growth traits therefore suggests the possibility of an exploration–exploitation trade-off in AM fungal growth strategy. Such a trade-off could arise from constraints due to a limiting nutrient resource. Unlike free-living filamentous fungi, AM fungi, as obligate biotrophs, rely entirely on their plant host for their carbon supply (22). Given that the trade-off itself was dependent on host identity, we suspected that host carbon and not other nutrients (nitrogen, phosphorus, or other macro/micronutrients) could be directly limiting. We therefore estimated how much carbon was expended by the fungal network on hyphal and spore growth at each timestep. We defined the rate of carbon expenditure $\Phi_{C}$ as

[2] $$\begin{array}{c} \Phi_{C}=\frac{dC_{\text{t}}}{\mathrm{dt}}\times \frac{1}{\mathrm{CUE}}, \end{array}$$

where CUE is carbon use efficiency i.e. the ratio of carbon incorporated in biomass (and not respirated) to all the carbon used for growth, a quantity often measured in ecology. We used CUE=50%, corresponding to an average for soil microorganisms such as soil fungi (23, 24). Eq. **2** is a leading-order approximation of the rate of carbon expenditure that neglects maintenance costs of the network ($\propto C_{t}$) as well as possible variations in CUE (*SI Appendix*, Fig. S4 and section 4.A).

We found that $\Phi_{C}$ increased substantially (by up to ∼10-fold) over time for all strains observed (Fig. 2*B*). Thus, if carbon is indeed the growth-limiting resource, this increase in $\Phi_{C}$ implies that carbon limitation of fungal growth is being progressively relaxed over time.

## Fungal P Supply Scales with Network Surface Area

**Fig. 3.**
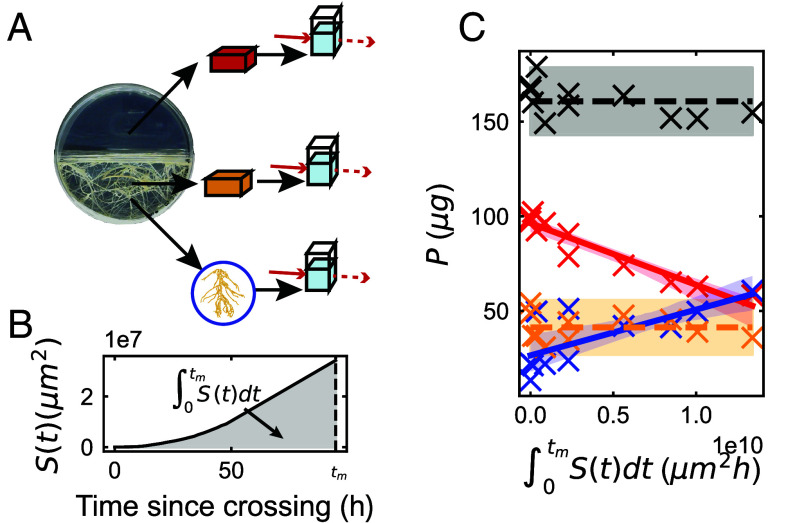
Phosphorus depletion and transfer by the growing network. (*A*) Illustration of the experimental method used for measurement of phosphorus mass in the fungal compartment (red), the gel of the root compartment (orange) and the host root (blue) (*B*) Network surface area S(t) (black curve) plotted up to harvest at time $t_{m}$ for a typical plate. Gray shaded area under the curve highlights the time-integrated surface area, a key quantity in our analysis. (*C*) Total mass of phosphorus measured in the fungal compartment ($P_{\text{f}}$, red), the gel of the root compartment ($P_{\text{g}}$, orange) and the host root isolated from the root compartment ($P_{\text{r}}$, blue) as a function of the time-integrated surface area. The black line and dots correspond to the total phosphorus $P_{\text{t}}$ across all three measurements. Points correspond to individual replicates, full lines correspond to linear fit, dashed horizontal lines show averages for the corresponding category. Shades of full lines correspond to the 95% C.I. of the linear fit obtained via bootstrapping and shades of dashed lines to (mean ±2× s.e.m.). Coefficient of determination for the linear fits (*R*^2^) are 0.91 (red), 0.51 (blue).

What could account for such a large increase in carbon input from the plant host during fungal network growth? Because AM fungi forage and exchange phosphorus to obtain plant carbon, we hypothesized that the evident increase in C supply from the plant host reflects a concomitant increase in P transfer to the plant root via the fungal network. To test this idea, we proceeded to quantify the amount of environmental P absorbed by the fungal network and transferred to the root. Using a spectrophotometric assay (*SI Appendix*, section B), we measured the total mass of phosphorus in the fungal compartment, the gel of the root compartment, and within the host root (Fig. 3*A*). Next, we estimated the total surface area of the network $S=\sum_{i\in edges}2\pi r_{i}L_{i}$ at each point in time until harvest using the network morphology extracted from our imaging pipeline (Fig. 3*B*).

Assuming the absorption of P by the fungal network is limited by the number of P transporters on the hyphal surface at some characteristic density (25), the rate of P absorption $\Phi_{P}$ will be a product of the total surface area of the network S and the rate of transport per unit area J. The depletion of total phosphorus in the fungal compartment $P_{\text{f}}$ due to absorption can then be expressed as,

[3] $$\begin{array}{c} \frac{dP_{\text{f}}}{\mathrm{dt}}=-JS, \end{array}$$

where the rate coefficient J may be expected to depend, as is typical for membrane transporters, on the extracellular concentration of P as $J\left(\left[P\right]\right)=J_{\text{max}}\frac{\left[P\right]}{\left[P\right]+K_{m}}$, where [P] is the P concentration and $K_{m}$ is a transporter-dependent parameter corresponding to the P concentration at which $J=J_{\text{max}}/2$ (*SI Appendix*, section 2.F.2). Thus, if [P] remains sufficiently high to saturate the transporters (i.e. $\left[P\right]\gg K_{m}$) throughout the duration of the experimental measurement, J would remain nearly constant at $J\approx J_{\text{max}}$ (*SI Appendix*, section 2.F.2) and we can integrate Eq. **3** up to the measurement time $t_{\text{m}}$ as,

[4] $$\begin{array}{c} P_{\text{f}}\left(t_{\text{m}}\right)=P_{\text{f}}\left(0\right)-J\int_{0}^{t_{\text{m}}}S\left(t\right)\;dt. \end{array}$$

which predicts that the amount of P left in the compartment $P_{\text{f}}$ will decrease linearly as a function of the time integrated surface area $\int_{0}^{t_{\text{m}}}S(t)dt$ with slope −J. We found that the measured $P_{\text{f}}$ did indeed decrease linearly with integrated surface area (Fig. 3*C*, red), and the slope allowed us to extract J≈3 ng·mm^−2^h^−1^. This transport parameter is important for the plant-fungal symbiosis because it determines the rate at which a given network will absorb P from its environment. For example, a network with this value of J, total length 1 m, and an average radius 3 μm would extract about 1 μg of P per day. In contrast to $P_{\text{f}}$, the amount of phosphorus $P_{\text{g}}$ in the gel of the root compartment [which represents a baseline amount of inaccessible phosphorus, presumably adsorbed to the gel matrix (26)] did not significantly change throughout network growth (Fig. 3*C*, orange).

Our estimate J≈3 ng·mm^−2^h^−1^ obtained by imaging network morphology and P depletion within in vitro networks is in agreement with the range of values obtained in previous studies (J≈4to10 ng·mm^−2^h^−1^) based on fungal density and P concentration measurements in soil (27, 28). We also found that the decrease in $P_{\text{f}}$ was balanced by a complementary increase in the amount of P in the host root $P_{\text{r}}$ (Fig. 3*C*, blue). Given that neither the amount of phosphorus $P_{\text{g}}$ in the gel of the root compartment (Fig. 3*C*, orange) nor the total amount of phosphorus $P_{\text{t}}$ ($=P_{\text{f}}+P_{\text{r}}+P_{\text{g}}$) across all three measurements (Fig. 3*C*, black) significantly changed in the meanwhile, the increase in $P_{\text{r}}$ can be wholly attributed to the symbiotic transfer of P across compartments via the fungal network and into the host root. Taken together, these data suggest that in these experiments, nearly all the P absorbed by the fungal network is efficiently transferred to the host root, without extensive obstructions or delays that would result in a build up of P inside the fungal hyphae in contrast to what had been observed in other studies (11, 29). This latter conclusion is further supported by considering our results in the context of literature reports of P concentration in AM fungal hyphae. The magnitudes of P absorption and network volume we measured suggest that approximately 30% of fungal dry biomass would be P if no P were transferred to the root (*SI Appendix*, Fig. S5). Yet across the literature, the highest reported mass fraction of phosphorus in AM fungal hyphae is 4% of total dry weight (12, 30, 31) while polyphosphate content in yeast does not seem to greatly exceed 10% (32). Taking 5% as a conservative estimate for the P-fraction of fungal total dry weight, this would mean at least 85% of all absorbed P would have been transferred to the plant after 100 h of growth. These observations highlight the remarkable efficiency of AM fungi in moving P resources—transferring an amount of P corresponding to nearly a third of their total dry weight to their plant host within ∼100 h.

In summary, we found that 1) nearly all of the P absorbed by the network is transferred to the root, and 2) the rate of absorption remains well approximated throughout the course of P depletion as proportional to hyphal network surface area S. That is, within these experiments,

[5] $$\begin{array}{c} \Phi_{P}\approx JS\approx \text{Rate of P transfer to root}, \end{array}$$

until P is completely depleted. Consequently, the model of Eq. **3**, calibrated with values of J obtained from our P-depletion measurements, provides a means to estimate $\Phi_{P}$ and the rate of P transfer to the root from imaging-based measurements of total network surface area S.

## Proportionality Between C Expenditure and P Supply Reveals a Plant-Dependent Exchange Rate

**Fig. 4.**
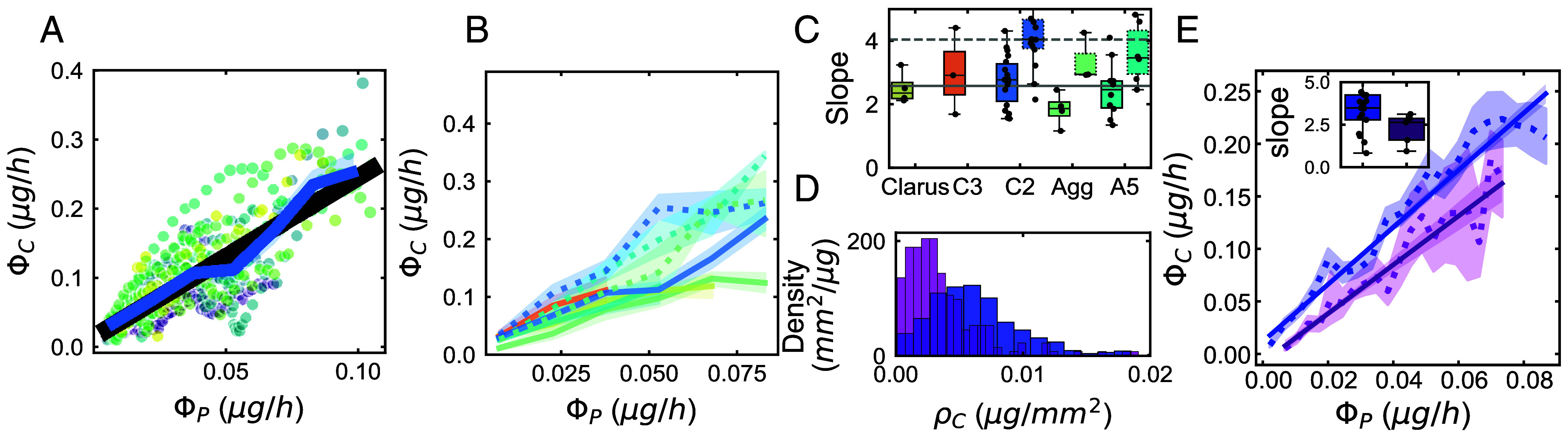
$\Phi_{C}/\Phi_{P}$ ratio depends on plant host but not P availability. (*A* and *B*) Carbon expenditure rate ($\Phi_{C}$) as a function of Phosphorus supply rate ($\Phi_{P}$). (*A*) *R. irregularis* strain C2 with genotype 2 carrot root. Each point corresponds to a measurement of $\Phi_{C}$ and $\Phi_{P}$ at one timestep for one replicate, each replicate is shown in a different color (n=18). The blue line shade corresponds to binned average 95% C.I. over regular $\Phi_{P}$intervals. The black line corresponds to linear fit over the blue points. (*B*) For *R. irregularis* A5 (cyan; *n_genotype 1_* = 11, *n_genotype 2 =_* 6), C2 (blue; *n_genotype 1_* = 18, *n_genotype 2_* = 11), C3 (orange, n=3), *R. aggregatus* (green; *n_genotype 1_* = 4, *n_genotype 2_* = 3), *R. clarus* (yellow, *n*= 4) associated with genotype 2 (dashed line) and genotype 1 (full line). Lines and shades correspond to the same calculations as in (*A*). (*C*) Slopes of the linear fit forced through the origin for each host-fungus genotype combination. Full boxes correspond to genotype 1 and dashed boxes to genotype 2. The gray line corresponds to the mean of all slopes for each genotype. Full line correspond to genotype 1 and dashed line to genotype 2. Number of replicates and color-coding is the same as in (*B*). (*D*) Distribution of instantaneous carbon density ($\rho_{C}$) at high (1.4 μg/mL, blue) and low (0.6 μg/mL, purple) available phosphorus concentrations in the fungal compartment for *R. irregularis* C2 grown with genotype 2 carrot root. Each count included in the bins corresponds to a measurement of $\rho_{C}$ at one timestep for one replicate. Number of replicates in the high- and low-P conditions were 18 and 7, respectively, totaling 722 and 209 counts, respectively. (*E*) $\Phi_{C}$ as a function of $\Phi_{P}$ for *R. irregularis* strain C2 networks grown at high (blue) and low (purple) P concentrations. Lines and points are as in (*A*). *Inset*: Slopes of the linear fit forced through the origin for each replicate for each condition. For all boxplots, the box represents the interquartile range (IQR), with the central line indicating the median. The whiskers extend from the box to the minimum and maximum values within 1.5 times the IQR.

Within our two-compartment petri dish setup, the fungus has access to C only through the plant host. Likewise, throughout the time course of our network imaging, the plant host has access to P only through the fungal network. We therefore reasoned that examining the relationship between the rate of phosphorus transfer ($\Phi_{P}$) and the rate of carbon expenditure ($\Phi_{C}$) at every time point could provide insight into the nature of nutrient exchange. Despite considerable variation at the individual plant-fungal replicate level, we found that the relationship between these two rates averaged across all replicates (Fig. 4*A*, blue curve) was well approximated by a straight line (Fig. 4*A*, black curve), with a slope of approximately 3 mass units of C per 1 mass unit of P. While the exact magnitude of the slope inherits aforementioned uncertainties in estimating CUE and $M_{C}$ [e.g. for CUE relative uncertainty is around 30% (23); see also *SI Appendix*, section 4.A], our main conclusion that the $\Phi_{C}/\Phi_{P}$ ratio is on average constant remains valid as long as the values of these parameters are invariant across time, space, and across treatments.

Because both the carbon expenditure rate $\Phi_{C}$ and phosphorus uptake rate $\Phi_{P}$ increase with network size, we asked whether this constant ratio might be a logical consequence of traveling-wave growth. To consider how $\Phi_{C}$ and $\Phi_{P}$ scale as the network expands its spatial range, we first identify three regimes of network growth (*SI Appendix*, section 3). The first regime occurs at early times (t≲50 h) before the hyphal tip- and filament-density dynamics develop into a traveling wave. The network exhibits exponential growth in this regime (17), which implies $\frac{\mathrm{dL}}{\mathrm{dt}}\propto L$ (*SI Appendix*, section 3.A). Given also that $\Phi_{C}\propto \frac{\mathrm{dV}}{\mathrm{dt}}\propto \frac{\mathrm{dL}}{\mathrm{dt}}$ and $\Phi_{P}\propto S\propto L$ (see above, and *SI Appendix*, section 3.A), this leads to the prediction that $\Phi_{C}\propto \Phi_{P}$. The second regime begins at a time $t_{0}$ (≈50 h) at which the hyphal density wavefront establishes, after which this wavefront travels at a constant speed. Using the radius $R_{\text{wave}}$ of its spatial range growing at a constant wave speed $v_{\text{wave}}$ ($R_{\text{wave}}\left(t\right)=v_{\text{wave}}t$), we derived analytically the following expressions for $\Phi_{C}$ and $\Phi_{P}$ (*SI Appendix*, section 3.B.2):

[6] $$\begin{array}{cc} \Phi_{C} & =2\pi \frac{\rho_{C}}{\mathrm{CUE}}v_{\text{wave}}R_{\text{wave}}+\Phi_{C}^{\left(S\right)} \end{array}$$

[7] $$\begin{array}{cc} \Phi_{P} & =J2\pi \rho_{S}R_{\text{wave}}\Delta R, \end{array}$$

where $\Phi_{C}^{\left(S\right)}$ is the carbon spent on spore production (not accounted by the traveling wave model), $\rho_{S}$ is the density of hyphal surface area and ΔR is the radial width of the annulus in which P is not yet depleted. The second regime persists as long as no region of space is yet depleted in P, and therefore $\Delta R=R_{\text{wave}}$. This means, using Eqs. **6** and **7**, that the ratio $\Phi_{C}/\Phi_{P}$ from the traveling-wave model alone (i.e. when $\Phi_{C}^{\left(S\right)}$ is excluded) would scale as $\Phi_{C}/\Phi_{P}∼R_{\text{wave}}^{-1}$ (*SI Appendix*, section 3.B.3). Thus, the observation of a constant $\Phi_{C}/\Phi_{P}$ ratio implies a significant carbon expenditure into spore production. This is in agreement with the observation that spore formation contributes ∼25% of carbon expenditure by around 100 h (*SI Appendix*, Fig. S4). The third regime begins at a time $t_{1}$ (≈8 d, *SI Appendix*, section 3.C) when a P-depletion front establishes and trails the advancing hyphal density wavefront at the same speed $v_{\text{wave}}$. In this regime, it can be shown that ΔR adopts a constant value (*SI Appendix*, section 3.C). As long as $\Phi_{C}^{\left(S\right)}\ll 2\pi \rho_{C}v_{\text{wave}}^{2}t$, the ratio $\Phi_{C}/\Phi_{P}$ settles again to a constant

[8] $$\begin{array}{c} \frac{\Phi_{C}}{\Phi_{P}}=\frac{\rho_{C}}{CUE{\left[P\right]}_{0}d}, \end{array}$$

where ${\left[P\right]}_{0}$ is the P concentration ahead of the front and d is the depth of the agar gel. However, the data of Fig. 4*A* extend only up to times (≤5 d), considerably earlier than the onset of such a steady state (≈8 d), and hence can not explain the constant $\Phi_{C}/\Phi_{P}$ ratio observed in our experiments

Taken together, these observations suggest that the observed proportionality between $\Phi_{C}$ and $\Phi_{P}$ throughout network development is not a trivial consequence of the traveling-wave growth. Furthermore, we found that the on-average linear dependence of $\Phi_{C}$ on $\Phi_{P}$ was recapitulated across all five tested AM fungal genotypes (Fig. 4*B* and *SI Appendix*, Fig. S6) and, moreover, the slopes of these dependences were approximately the same (Fig. 4*C*) despite wide variation in carbon density of the wave $\rho_{C}$ and its propagation speed $v_{\text{wave}}$ (Fig. 2*A*). This invariance of the $\Phi_{C}/\Phi_{P}$ ratio over time and across genotypes suggests the existence of one or more mechanisms that regulate fungal growth under symbiotic resource exchange.

By contrast to the near-invariance across fungal genotypes, we found a clear change in the $\Phi_{C}/\Phi_{P}$ ratio upon changing the genotype of the plant host (Fig. 4 *B* and *C*). When grown with host genotype 2 (i.e. fast growing root), the slope of the dependence of $\Phi_{C}$ on $\Phi_{P}$ increased for all three tested fungal genotypes (*R. irregularis* A5, *R. irregularis* C2, *R. aggregatus*) compared to when grown with host genotype 1. And similarly to when grown with genotype 1, these slopes across the three fungal strains were remarkably similar, despite the stark contrasts in their growth traits ($\rho_{C}$, $v_{\text{wave}}$) when grown with either hosts (*SI Appendix*, Fig. S3). Thus, while the $\Phi_{C}/\Phi_{P}$ ratio remains constant across fungal strains when attached to the same plant host, it can vary in a manner that depends on the plant host genotype.

If a change in the plant host (which supplies C) can vary the $\Phi_{C}/\Phi_{P}$ ratio, could varying the availability of P also have an effect? To answer this question, we repeated the experiments with different amounts of P in the fungal compartment. Our hypothesis was that, because of the reciprocal nature of exchange, lower P availability would result also in a reduced C supply from the plant, thereby rendering the C-limitation of growth more stringent. The fungus could then respond by decreasing the saturating carbon density $\rho_{C}$, the propagation speed $v_{\text{wave}}$ of its traveling-wave growth, or both.

We reduced the amount of soluble P available for fungal absorption ${\left[P\right]}_{0}$ to approximately one third and found that the fungal density $\rho_{C}$ was substantially reduced, by approximately two-fold (Fig. 4*D* and *SI Appendix*, Fig. S3). By contrast, we found the wave speed $v_{\text{wave}}$ was barely affected by this reduction in [P] (*SI Appendix*, Fig. S3). These effects on the traveling-wave parameters can be understood through Eqs. **6** and **7** and the hypothesis of a fixed ratio $\Phi_{C}/\Phi_{P}$. At sufficiently low initial P concentration ${\left[P\right]}_{0}$, it is expected that a totally P-depleted zone (where [P]→0) would form in the inner regions of the wave, leading to a relative narrowing of the P-depleted zone ($\Delta R<R_{\text{wave}}$) and a consequent decrease in $\Phi_{P}$ compared to the higher ${\left[P\right]}_{0}$ condition (where $\Delta R=R_{\text{wave}}$). At fixed $\Phi_{C}/\Phi_{P}$, that reduction in $\Phi_{P}$ would in turn lead to a decrease of $\Phi_{C}$, which according to Eq. **6** can be achieved by decreasing $\rho_{C}$. To test this hypothesis, we adapted our method of computing $\Phi_{P}$ to account for potential P depletion (*SI Appendix*, section 2.F.2). Remarkably, despite the reduction in carbon flux, the relationship between $\Phi_{C}$ on $\Phi_{P}$ remained well-approximated as a straight line, with nearly the same slope as the control condition with threefold higher ${\left[P\right]}_{0}$ (Fig. 4*E*). This invariance in the $\Phi_{C}/\Phi_{P}$ ratio motivates a parsimonious explanation for the observed reduction in $\rho_{C}$: it reflects the response of the fungus to a reduced C budget, which in turn results from the attenuated P supply for obtaining C through reciprocal exchange.

Overall, these data are consistent with a mode of reciprocal C/P exchange in which C supply from the plant host $\Phi_{C}$ is approximately proportional to P supply from the fungus $\Phi_{P}$. The constant of proportionality $\Phi_{C}/\Phi_{P}$ can be considered the exchange rate for reciprocal trade and appears invariant under changes in AM fungal genotype and P available to the fungal network but can depend on plant host genotype.

## P Absorption Shapes Network Growth Phenotypes and Symbiotic Outcome

Can the observed proportionality of reciprocal resource exchange help us understand other salient features of AM fungal behavior, and, in turn, how these features affect symbiotic outcomes? To answer this question, we extended a recently proposed model of fungal traveling-wave range expansion (17) to couple network growth to resource exchange. The model describes the coupled dynamics of growing hyphal tips and hyphal filaments. The spatial density of growing tips are represented by their spatial density n(R,t) (with units mm^−1^), and that of hyphal filaments by ρ(R,t) (with units µm·mm^−1^) along the radial coordinates R. The density of hyphal filament length ρ is governed by the density of tips, which elongate the filaments at growth speed $v_{\text{g}}$ to densify space,

[9] $$\begin{array}{c} \frac{\partial \rho }{\partial t}=v_{\text{g}}n. \end{array}$$

The density of growing tips n is governed by branching at rate αn that gives rise to new tips, anastomosis at a rate βnρ that annihilates tips upon collision with existing filaments, and their motion in space (due to growth and branching) that can be described by a density-dependent spatial flux j(n),

[10] $$\begin{array}{c} \frac{\partial n}{\partial t}=\alpha n-\beta n\rho -\nabla \cdot j\left(n\right). \end{array}$$

This model has traveling-wave solutions with a constant wave speed $v_{\text{wave}}$ that is determined by model parameters that are determined by underlying statistics of growth and branching (17) and *SI Appendix*, section 2.G.1].

The model solutions for ρ(R,t) and $v_{\text{wave}}$, combined with Eqs. **6** and **7**, allow $\Phi_{C}$ and $\Phi_{P}$ to be computed for all points in time during traveling-wave growth (*SI Appendix*, section 2.G.2). However, we note that the model fails to capture two key features of our experimental findings on $\Phi_{C}$ and $\Phi_{P}$, namely 1) the ratio $\kappa =\Phi_{C}/\Phi_{P}$ remains constant throughout the course of network growth, and 2) faster expanding strains tend to invest more in wider hyphae (*SI Appendix*, Fig. S7), a relationship found also in other filamentous fungi (33). We therefore sought to extend the traveling-wave model to account for both of these features 1) and 2).

As a minimal extension to the model to maintain the $\Phi_{C}/\Phi_{P}$ ratio κ constant (at the value of the exchange rate $\kappa_{0}$), we introduced a κ-dependent feedback on the branching rate α,

[11] $$\begin{array}{c} \frac{d\alpha }{\mathrm{dt}}=F\left(\kappa \right), \end{array}$$

where the function F(κ) can be chosen to implement negative integral feedback—a robust control engineering strategy (34) widely found also across biological systems (35–37)—so that the feedback variable α decreases when $\kappa >\kappa_{0}$ and increases when $\kappa <\kappa_{0}$, so as to maintain the output variable κ nearly constant ($\kappa \approx \kappa_{0}$) at all times (*SI Appendix*, section 2.G.3).

To model the observed dependence of hyphal width on network expansion speed, we assumed the simplest affine form for the functional dependence of the average hyphal radius ⟨r⟩ on wave speed, $\left\langle r\right\rangle =av_{\text{wave}}+b$, with parameters a and b obtained by fitting the data in *SI Appendix*, Fig. S7.

**Fig. 5.**
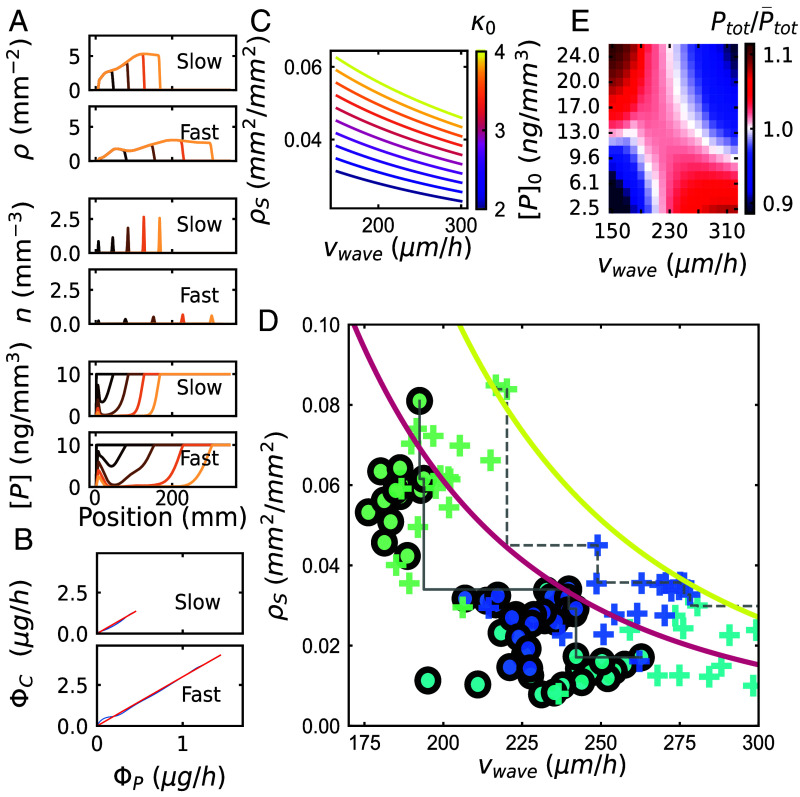
Extended traveling-wave model reveals an exchange-rate-dependent Pareto front. (*A*) Numerical simulation results with $v_{\text{wave}}=150\;\mu$m/h (*Upper*), and with $v_{\text{wave}}=300\;\mu$m/h (*Lower*): Color gradients correspond to sampling at regular time intervals, from *t*= 0 h to *t*= 900 h. The first row is the hyphal length density ρ, the second row is the tip density n and last row is the P concentration [P]. (*B*) $\Phi_{C}$ plotted against $\Phi_{P}$, during the same simulations. The red line corresponds to $\Phi_{C}$= $\kappa_{0}\Phi_{P}$, the blue line corresponds to model outputs. *Upper* and *Lower* are as in (*A*). (*C*) Family of “isoexchange rate” curves computed from the analytical approximation of the traveling-wave model in the long-time limit (*SI Appendix*, section 3.C.3) also refer to Eq. **12**, indicating a trade-off between surface area density $\rho_{S}$ and wave speed $v_{\text{wave}}$. These curves bound from above the range of achievable AM fungal growth strategies constrained by the value of the fixed exchange rate, $\kappa_{0}$ (indicated by color). (*D*) Experimentally observed phenotypes in $\rho_{S}$-$v_{\text{wave}}$ space. Data points represent median values over successive 10-h intervals in each plate, with color code as in Fig. 4. Circles, to host genotype 1; crosses, to host genotype 2. Gray lines, moving maximum of $\rho_{S}$ for decreasing values of $v_{\text{wave}}$ for each host genotype (full: genotype 1, dashed, genotype 2). Colored curves, predicted Pareto fronts computed from the analytical approximation for intermediate times when the P depletion front has yet to fully develop (*SI Appendix*, section 3.D) for $\kappa_{0}=3$ and $\kappa_{0}=4$ [color code as in (*C*)]. (*E*) $P_{\mathrm{tot}}/{\overset{¯}{P}}_{\mathrm{tot}}$ as a function of wave speed *v_wave_* and initial concentration ${\left[P\right]}_{0}$. Color corresponds to the relative increase in total P absorption $P_{\mathrm{tot}}$ compared to the average ${\overset{¯}{P}}_{\mathrm{tot}}$ across each row corresponding to a given value of ${\left[P\right]}_{0}$ (*SI Appendix*, section 2.H.5).

To test the extended model, we parameterized it with measured network-growth data and examined the predicted patterns of growth. Parameter sets for slower expanding strains (Fig. 5*A*, *Upper* panels) yielded denser growth than those for faster expanding strains (Fig. 5*A*, *Lower* panels), while the rate of phosphorus transfer ($\Phi_{P}$) and the rate of carbon expenditure ($\Phi_{C}$) were indeed maintained approximately proportional throughout (Fig. 5*B*). These results suggest that the apparent trade-off we observed between network density and expansion speed (Fig. 2*A*) could be explained by traveling-wave migration under the constraint of a fixed C/P exchange rate and expansion speed-dependent hyphal thickness.

Consistent with this idea, adapting the relationship of Eq. **8** (*SI Appendix*, section 3.C.3) yields a family of “isoexchange rate” curves linking the position of fungal phenotypes that match a fixed ratio $\kappa_{0}=\frac{\Phi_{C}}{\Phi_{P}}$. Those can be placed in a $\rho_{S}$-$v_{\text{wave}}$ space where the $\rho_{S}$ axis represents the hyphal surface area density (Fig. 5*C*). The curves are parameterized by the following relationship

[12] $$\begin{array}{c} \rho_{S}=\kappa_{0}\frac{CUE{\left[P\right]}_{0}}{M_{C}\pi \xi \left(av_{\text{wave}}+b\right)}, \end{array}$$

where ξ is a constant that depends on the coefficient of variation of the radius distribution (*SI Appendix*, section 2.G.5).

These curves can be considered Pareto fronts (9) delineating the limits of achievable traveling-wave growth strategies under the constraint of fixed exchange rate. Their negative slope indicates a trade-off—increasing expansion speed (favoring exploration) tends to decrease growth density (favoring exploitation), and vice versa—and their convex shape suggests a drive toward specialization—fungi can explore fast, or exploit fast, but not both. Interestingly, as the C/P exchange rate $\kappa_{0}$ is increased, the Pareto front shifts upward, thereby broadening the range of accessible strategies. Indeed, plotting our experimental data (*SI Appendix*, Fig. S3) in $\rho_{S}$-$v_{\text{wave}}$ space revealed a shift in the apparent Pareto front upon swapping the plant host and therefore increasing the exchange rate (Fig. 5*D*). Finally, the same model could also explain the decreased fungal density at lower P availability (Eq. **12**), consistent with our experimental observations (Fig. 4*C*). We therefore conclude that the extended traveling-wave model with exchange-rate feedback and speed-dependent hyphal widths captures salient patterns of fungal phenotype distributions and their dependence on environmental conditions.

What are the functional implications of this trade-off? Whereas fast network expansion (high $v_{\text{wave}}$) promotes exploration for new resources and trade partners, a higher network density allows for faster exploitation of local resources (because $\Phi_{P}$ scales with the network surface area density $\rho_{S}$, which in turn is proportional to network length density ρ). The observed density–speed trade-off can therefore be interpreted as an exploration–exploitation trade-off: fungal phenotypes that prioritize distal exploration will tend to receive less carbon in the short term, whereas those that rapidly exploit local resources will tend to be slower at discovering new resources and trade opportunities.

We further asked whether and how this exploration–exploitation trade-off could also explain environmental context dependence of the AM symbiosis. We addressed this question by simulating fungal network growth under different combinations of initial P concentration ${\left[P\right]}_{0}$ and fungal growth strategy $v_{\text{wave}}$. For each initial concentration, we compared the total amount of P transferred during a fixed interval of time $P_{\mathrm{tot}}$ to its average ${\overset{¯}{P}}_{\mathrm{tot}}$ over all strategies. The resulting ratio is a measure of P absorption performance and hence reflects also the comparative benefits of different fungal strategies for P supply to the plant. We found that the wave speed that provides the highest relative phosphorus transfer $P_{\mathrm{tot}}/{\overset{¯}{P}}_{\mathrm{tot}}$ depended on ${\left[P\right]}_{0}$ (E). Specifically, at higher ${\left[P\right]}_{0}$, slow-expanding networks that invest in dense growth without paying extra costs can better capture the available P. By contrast at low ${\left[P\right]}_{0}$, fast-expanding networks can better escape their self-generated P-depletion zone (Fig. 5*A*, *Bottom* panels), leading to an overall advantage in capturing P.

These results demonstrate how the P absorption and transfer performance of AM fungi, which often determines observable symbiotic outcomes such as plant growth (38), can strongly depend on the context of symbiosis, such as the plant-fungal genotype combination (which strongly affects $v_{\text{wave}}$) and environmental conditions such as the accessible P concentration.

## Discussion

By leveraging robotic imaging combined with machine learning, we developed an approach to estimating resource fluxes in the mycorrhizal symbiosis based on in toto quantification of network morphology throughout growth. We identified a systematic proportionality between carbon transfer from the plant host and phosphorus transfer by the AM fungal network (Fig. 4). We found that the proportionality coefficient remained constant across various fungal strains associated with the same plant host, despite wide variation in their growth phenotypes. Collectively, our results suggest that fungal network growth is constrained not by carbon supply per se, but rather by the exchange rate of reciprocal C/P exchange, which determines the amount of carbon that the fungus can obtain per unit of collected phosphorus.

Previous studies that used isotope tracers in similar in vitro root organ cultures (11) and in real soils (15) obtained compelling evidence for reciprocal exchange, but whether and to what extent the exchange is proportional has remained challenging to determine. In in vitro cultures, it was found that more host-derived carbon was transferred to fungal compartments where a greater quantity of P was available (11) and that fungi partners accumulate more nutrients in hyphae and spores when associated with a plant that has less access to carbon (12). In real soils, quantitative radiotracer counting showed a positive correlation between P delivery to the plant and host C allocation to the AM fungal partner (15). However, it has also been suggested that P supply may not always be tied directly to carbon allocation. Specifically, C surplus theories hypothesize that the amount of carbon transferred to mycorrhizal fungi is independent of nutrient return from the fungus (13) and only depends on plant metabolism and fungal “carbon sink strength.” Our present work demonstrates that fungal “carbon sink strength” is not fixed by the size of the network. Instead, both range-expansion speed and saturating network density affect how much carbon the fungal network consumes at a given point in time (Fig. 2 and *SI Appendix*, Fig. S3). We also found a trade-off between network density and expansion speed. This is important because it suggests that the fungal partner is constantly constrained by carbon exchange, such that the same network cannot be both dense and fast.

Our finding that the C/P exchange rate depends on the host plant genotype raises a wealth of interesting questions. How might this exchange rate vary with various environmental conditions of the host? And might the rate vary if the plant were trading with not one, but multiple fungal partners simultaneously? More generally, what are the factors that set the exchange rate, and through what mechanism do they act? On the host side, control of arbuscule formation (39) or lifetime (40, 41) have been suggested as potential mechanisms to control carbon flux into AM fungal networks. The necessity for control of lipid biosynthesis and export to the AM partner may also require complex feedbacks involving regulation of gene networks (42). In the case of the legume-rhizobium symbiosis, theoretical and experimental exchange rate of similar order of magnitude to ours (3 to 10 C/N vs. 3 to 4 C/P) have been reported (43, 44). In those cases, the exchange rate could be inferred from the metabolic cost of nitrogen fixation while we found that in the case of the plant-mycorrhizal symbiosis, it can be determined from the spatial dynamics of fungal propagation and P depletion (Eqs. **6** and **7**).

The context dependency of the plant-mycorrhizal symbiosis has been the focus of research for decades (3–5, 45–48). Despite this growing body of work, predicting the nutrient-exchange outcomes of the symbiosis has remained a puzzle. Our experimental findings and theoretical model provide a foundation for exploring how proportionality of resource exchange affects total symbiotic outcomes, such as P transfer from AM fungi to plants, across varying contexts and hosts.

Our modeling framework could also help understand how changes in the mobility of nutrients such as P, due e.g. to layered soil texture or chemical properties, can affect the optimal range-expansion strategy. Typically, root systems are simultaneously colonized by multiple AM fungal partners (49, 50). It would also be straightforward to extend our modeling framework to include multiple species with contrasting range-expansion strategies, to address the stability of coexistence (51). An important question is how a diversity of partners with complementary traits could affect P transfer to plants, and whether the proportionality documented here changes as the number of partners change (14). Exploring these questions could help explain why so many AM fungal species can coexist within the same niche, a long-standing question in AM ecology (52).

Future work is needed to simultaneously characterize these traits across fungal strains found in different environments, as well as quantifying the total amount of P transferred to plants. For example, in previous pot experiments grown under different P fertilization treatments, *R. irregularis* provided more P to the host plant compared to *R. aggregatus*. However, the difference between the two strains became insignificant with higher P fertilization (53). Following the temporal dynamics of these differences, and how they coincide with amount, location and regularity of fertilizer applications is a next important step.

Our work demonstrates the functional significance of subtle, micron-scale traits, such as the hyphal radius distribution, in understanding symbiotic resource exchange. We initially suspected that thinner structures would be favored for resource absorption. This is because the carbon cost of a hypha grows as the square of its radius, while its ability to absorb nutrients from the environment grows only linearly. The most cost-efficient absorbing structures therefore are the thinnest ones. This principle is exemplified in plants where root hairs are thinner than main roots, and also in AM fungi where branched absorbing structures (BAS) (54) tend to be the thinnest of all hyphae across the AM network (Fig. 1*C*). However, our findings on how hyphal radius relates to other growth phenotypes indicates that minimizing the cost of building absorption structures is not the only optimization objective. We found that faster expanding networks tend to invest more in thicker hyphae (*SI Appendix*, Fig. S7). This suggests that fast range expansion requires investment in thicker runner hyphae to allow the transport of C to the network periphery, as well as the transfer of P back to the host root. Our observation that AM fungi can change their hyphal width over time (Fig. 1*E*) further raises the possibility that hyphal width is a dynamically regulated morphological trait—perhaps to enhance the efficiency of long-distance nutrient transport necessitated by their symbiotic lifestyle. Fungal hyphae are generally believed to grow only axially, by elongation at their tips, thereafter maintaining an approximately constant hyphal width (55, 56) unless subjected to (e.g. osmotic) stresses (57). Evidently, AM hyphae also grow radially, by expanding the width of already built hyphal tubes over time. It is unknown if such radial growth is specific to AM fungi that have specific needs for long range transport or if other fungal hypha also have similar dynamics.

Because our data were gathered under sterile and in vitro laboratory conditions, it remains unknown whether, and to what extent, the observed proportionality of reciprocal exchange will extend to symbioses under natural conditions in which hyphae have their own microbiome that contribute to nutrient uptake (58, 59). It is also well known that AM fungi provide plants with a diversity of other benefits, including water, nitrogen, protection from pathogens, etc. (60). Based on our data, we would anticipate that benefits (e.g. nitrogen) that rely on network morphology, such as the total absorption surface area, might also yield similar proportionalities in the pattern of exchange. By using a similar approach to our phosphorus measurements, other nutrient exchanges of the symbiosis could be tested. Density and wave speed can also be important traits for the protection against pathogens because of competition for nutrients (61). Dense mycelium could effectively create a nutrient depletion barrier around the root that would make the host less accessible to pathogens.

In summary, our results identify the carbon–phosphorus exchange rate as a key constraint on AM fungal growth and range-expansion strategies. We found AM fungal genotypes were not positioned arbitrarily in the space of all possible strategies, but within a subregion bounded by an exchange-rate-dependent Pareto front (Fig. 5*D*). The shapes of the observed Pareto fronts suggest that fungal range-expansion strategies trade off exploration and exploitation performance under a given exchange rate. In turn, how fungal strategies position themselves within this phenotype space impacts P-transfer outcomes to the host plant. These observations provide a foundation for a mechanistic understanding of the stability of the mycorrhizal symbiosis and also for predicting coupled P and C dynamics at the earth system scale. Finally, the mass exchange ratio of ≈3 C/P implies that the flux of carbon in one direction and that of phosphorus in the other are of a similar order of magnitude. Maintaining mass fluxes of comparable magnitudes in opposite directions throughout the AM fungal network must require sophisticated strategies for routing resources throughout the network and perhaps even at the level of individual hyphae. How these dedicated symbionts manage their “supply-chain dynamics” for reciprocal nutrient exchange is a promising direction for future investigations.

## Materials and Methods

### Sample Preparation.

#### General recipe.

We used modified Strullu–Romand (MSR) medium (62) solidified with (3g/L) Phytagel and containing 10g/L sucrose (Carl Roth). The MSR medium in this study contained final concentrations of 739 mg/L MgSO_4_.7H_2_O, 76 mg/L KNO_3_, 65 mg/L KCl, 4.1 mg/L KH_2_PO_4_, 359 mg/L Ca(NO_3_)_2_.4H_2_O, 0.9 mg calcium pantothenate, 1 μg/L biotin, 1 mg/L nicotinic acid, 0.9 mg/L pyridoxine, 0.4 mg/L cyanocobalamin, 3 mg/L glycine, 50 mg/L myo-inositol, 1.6 mg/L NaFeEDTA, 2.45 mg/L MnSO_4_.4H_2_O, 0.28 mg/L ZnSO_4_.7H_2_O, 1.85 mg/L H_3_BO_3_, 0.22 mg/L CuSO_4_.4.5H_2_O, 2.4 μg/L Na_2_MoO_4_.2H_2_O, and 34 μg/L (NH_4_)Mo_7_O_24_.4H_2_O.

In the root compartment, the phosphate content of the medium was reduced to 1% of the above-mentioned concentration (41 μg/L KH_2_PO_4_) to stimulate mycorrhizal colonization of the roots. In the fungal compartment, we adjusted the phosphorus content in the MSR medium to impose low-P and high-P conditions: We applied either a high-P condition using the full MSR medium as described above, or a low-P condition in which KH_2_PO_4_ was removed entirely.

#### Low- and high-P conditions.

Specifically, for the low-P condition, we excluded KH_2_PO_4_ from the medium. Phytagel, the gelling agent used in this study, however, also contains P in a concentration that could not be modified. In preliminary experiments using Spectrophotometric assays (*SI Appendix*, section 2.B), we estimated the concentration of P in the phytagel to be 27 μmol/g. The fungal compartment was composed of 28 mL of MSR medium. Based on the above concentrations, this means the fungal compartment contained 96 μg P in the high-P condition and 70 μg P in the low-P condition corresponding respectively to concentrations of 3.4 μg/mL and 2.5 μg/mL.

We observed that the concentration of P in the root compartment did not seem to significantly decrease over time (*SI Appendix*, Fig. S3*B*). Also, observations over longer timescales (≥10 d) showed that the concentration in the fungal compartment seemed to plateau around similar but slightly higher value as the root P concentration after its initial decrease. P is known to be bound/adsorbed by soluble aluminum, iron, and manganese at low pH (63). pH in our plate was around 5, suggesting that potentially a large proportion of the P we could measure was not available to the plant or fungus. The slightly lower plateau concentration in the root compartment could be due to a change in pH due to the root presence. Based on measurement of root compartment plateau P concentration and fungal compartment plateau concentration at time ≥10 d, we estimated that 2 μg/mL of P were inaccessible. 0 concentration of P therefore corresponds to 0 concentration accessible by fungus and root but 2 μg/mL of P measured with our method. Concentration accessible by the fungus is therefore 1.4 μg/mL in the high-P condition and 0.5 μg/mL in the low-P condition.

#### Fungal strain.

All experiments were performed with *Rhizophagus irregularis* strains DAOM664344 (A5), DAOM664346 (C2), and C3 (Ian Sanders’ lab, Lausanne, Switzerland), *R. aggregatus* strain 0165, and *R. clarus* (Ian Sanders’ lab, Lausanne, Switzerland). All AM fungi were cultivated on regular MSR medium associated with Ri T-DNA transformed carrot root for 2 to 6 mo until plates were fully colonized.

#### Root strain.

All experiments were performed with two different root organ cultures of Ri T-DNA transformed carrot root. Ri T-DNA transformed carrot root “EU” (referred to as genotype 1 in main text) originated from a lab in Europe (see ref. 17). Ri T-DNA transformed carrot root “CA” (referred to as genotype 2 in main text) was *Daucus carota* subsp. Sativus, Cultivar enterprise, and originated from Canadian Collection of Arbuscular Mycorrhizal Fungi and was derived from Clone P68.

### Estimation of Hyphal Radius from Low-Resolution Images.

#### Label dataset generation.

Fungal networks grown in control conditions were imaged in the timelapse imaging system every 2 h [see *SI Appendix*, sections 2.A and 2.C (17) for details]. Immediately following imaging, we transferred the plates to the high-resolution imaging microscope. We then imaged locations across the network and recorded their network position in high resolution (i.e. 50X, see *SI Appendix*, section 2.D). We focused by positioning the optical plane in the center of the hypha. This was done on the basis that the cell wall should look thin and the active flows in the interior should be the widest. For each plate, we were able to accomplish this in less than 2 h. Images were then labeled using the software labelme. We drew three lines across the hypha that would span its whole external diameter. This external diameter included the hyphal cell wall. Using past experience, it was possible to establish what part of the imaged hypha corresponded to the interior with active flows and what could be attributed to the dark thick cell wall. In general, the dark thick cell wall never contributed more than 10 to 20% of the total diameter, which is in line with previous estimates of hyphal cell wall thickness for AMF (54). For each hyphal segment, we assigned the diameter corresponding to the mean of the three measurements. By repeating several independent measurements along several edges on these high-resolution image, we estimated the SD of the manual estimate to be of 0.3μm. This variation was either due to the difference in appreciation of the exact boundaries of the hyphae on the high-resolution image or to effective biological variation of the hyphal radius along an edge in a single field of view.

#### Feature dataset generation.

Next, we segmented and skeletonized the networks as in ref. 17. For each edge that had been imaged in higher magnification and labeled, we manually identified the corresponding edge of the network. We generated transects perpendicular to the skeleton and of size 120 pixels with the function profile_line of the package skimage (64). We selected the transect that was the closest to the position of the labeled image to use as a feature.

#### Data augmentation.

For part of the dataset, we varied the focus over 0.1 to 0.2mm above and below the “optimal focus” for images and varied illumination. Each label from high magnification was then associated with multiple features. In some other datasets, we extracted multiple transects close to the high magnification labeled image and associated them with the same label. This data augmentation procedure was done to help the model generalize. We also added transect corresponding to regions of the image empty of any hypha and a zero radius corresponding values in order to limit the overestimation of thinner hyphae.

#### Final dataset.

The final dataset, following data augmentation, consisted of 3,182 elements, which were divided into training, validation, and testing sets. Specifically, 90% of the data was used for training and validation, while the remaining 10% was kept for final testing. The test set consisted in a combination from entirely independent timesteps and plates from the rest of the dataset. We resampled the test set so it would follow the same distribution as the training set. To do so, we calculated the frequency distribution over 20 bins of the training set and sampled with replacement from the full test set. The distributions of the training/validation and resampled test sets are shown in *SI Appendix*, Fig. S1 *A* and *C*. Values ranged from 0 to 8 in both cases.

## Supplementary Material

Appendix 01 (PDF)

## Data Availability

Coarse-grained data tables summarizing network observables have been deposited in GitHub (https://github.com/Cocopyth/PC_2025). P extraction data shown in Fig. 3*C* were previously used under a different form in a previous published article. The analysis shown here is however fully different and is built upon newly extracted radius data (17).
