# Supplementary material for: Carbon–phosphorus exchange rate constrains density–speed trade-off in arbuscular mycorrhizal fungal growth
Source: Proc Natl Acad Sci U S A. 2026 Feb 6;123(6):e2512182123. doi: 10.1073/pnas.2512182123 (PMC12891005; doi:10.1073/pnas.2512182123)
Supplement: Supplementary file 1 — Appendix 01 (PDF) [file pnas.2512182123.sapp.pdf]

Supporting Information for

Supporting information: *Carbon-phosphorus exchange rate constrains density-speed tradeoff in arbuscular mycorrhizal fungal growth*

Corentin Bisot, Loreto Oyarte Galvez, Felix Kahane, Marije van Son, Bianca Turcu, Rob Broekman, Kai-Kai Lin, Paco Bontenbal, Max Kerr Winter, Vasilis Kokkoris, Stuart A. West, Christophe Godin, E. Toby Kiers, Thomas S. Shimizu

Thomas S. Shimizu, E. Toby Kiers, Corentin Bisot
E-mail: shimizu@amolf.nl, toby.kiers@vu.nl, bisot@amolf.nl

This PDF file includes:

Supporting text
Figs. S1 to S9
Tables S1 to S2
SI References

Supporting Information Text

Contents

1 Table of mathematical notations used in Main Text **3**

2 Supplementary Methods **3**

A Plate preparation details . . . 3
A.1 Assembly of Mesh Frames . . . 3
A.2 Two-compartment Split Plate Preparation . . . 4
A.3 Inoculation . . . 4
A.4 Plate Maintenance . . . 4
B Phosphorus Measurements . . . 4
B.1 Agar and root preparation . . . 4
B.2 Digestion . . . 4
B.3 Spectrophotometric determination of phosphate content . . . 4
C Timelapse imaging and network segmentation . . . 4
C.1 Label and feature dataset generation . . . 4
C.2 Network architecture and training procedure . . . 5
C.3 Evaluation . . . 5
D High resolution imaging . . . 5
E CNN model training and architecture . . . 5
E.1 Hyperparameter tuning . . . 5
E.2 Final architecture . . . 6
E.3 Training . . . 6
E.4 Evaluation . . . 7
E.5 Results . . . 7
F Carbon cost and phosphorus absorption calculations from data . . . 7
F.1 Computing carbon density and rate of carbon expenditure . . . 7
F.2 Computing P flux into the network . . . 8
G Model of network propagation with feedback . . . 9
G.1 General Framework . . . 9
G.2 Carbon Cost and Network P Absorption from model . . . 9
G.3 Exchange rate driven adaptation . . . 10
G.4 Rate of P absorption and P dynamics in the medium . . . 10
G.5 Accounting for hyphal radius differences . . . 10
H Numerical simulations . . . 11
H.1 Flux term . . . 11
H.2 Initial condition . . . 11
H.3 Other parameters . . . 11
H.4 Integration . . . 11
H.5 Fig. 5E . . . 11

3 Scaling of Φ_C and Φ_P across travelling wave growth regimes **11**

A Early-time regime: exponential hyphal network growth . . . 12
B Intermediate-time regime: Travelling-wave growth prior to P depletion . . . 12
B.1 Crossover time t_0 defining the onset of travelling-wave growth . . . 12
B.2 General derivation of Φ_C and Φ_P for a hyphal network travelling-wave . . . 12
B.3 Scaling of the Φ_C/Φ_P ratio . . . 12
C Late-time regime: Travelling-wave growth with a trailing P-depletion front . . . 13
C.1 Crossover time t_1 defining the onset of P-depletion front . . . 13
C.2 Expression for expected constant Φ_C/Φ_P ratio . . . 13
C.3 Iso exchange rate curves (Main Text Eq. 8) . . . 13
D Fitting of Pareto front (Fig. 5D) . . . 13

4 Supplementary discussion **14**

A Limitations of carbon estimates . . . 14
B Limitations of phosphorus estimates . . . 15
C Analytical considerations on the optimal wave speed . . . 15
C.1 Low P, Permanent regime . . . 16
C.2 High P, Exponential regime . . . 16
C.3 Optimal propagation in the non P depleted travelling wave regime . . . 16

5 Supplementary table 17

6 Supplementary figures 18

7 SI References 28

1. Table of mathematical notations used in Main Text

Table S1. Table of mathematical notations used in the manuscript

Symbol	Description	Unit
r	Hyphal radius (measured for each edge/segment).	μm
L	Total hyphal length of the network (sum over all segments).	mm
v_{wave}	Speed of the travelling-wave front of network expansion.	$mm\,h^{-1}$
C_t	Total carbon mass contained in the fungal network (hyphae+spores).	μg
$\rho_C = \frac{dC_t}{dA}$	Spatial carbon density: carbon per unit area of the expanding front.	$\mu g\,mm^{-2}$
Φ_C	Instantaneous carbon-expenditure rate (carbon "spent") by the network.	$\mu g\,h^{-1}$
M_C	Mass of carbon per unit cellular volume.	$\mu g\,mm^{-3}$
$V_i = \pi\, r_i^2\, L_i$	Volume of a cylindrical hyphal segment i with radius r_i and length L_i.	μm^3
$V_k = \frac{4}{3}\pi\, r_k^3$	Volume of a spherical spore k with radius r_k.	μm^3
CUE	Carbon-use efficiency (fraction of taken-up carbon incorporated into biomass).	dimensionless
$S = \sum_i 2\pi\, r_i\, L_i$	Total lateral surface area of the network (sum over all segments).	μm^2
P_{f}	Total phosphorus mass present in the fungal compartment.	μg
J	Phosphorus-absorption rate per unit hyphal surface area.	$ng\,mm^{-2}\,h^{-1}$
P_{g}	Total phosphorus mass present in the gel of the root compartment.	μg
P_{r}	Total phosphorus mass present in the root.	μg
$P_{\text{t}} = P_{\text{r}} + P_{\text{g}} + P_{\text{f}}$	Total measured phosphorus mass.	μg
Φ_P	Instantaneous phosphorus-transfer rate from the network to the plant.	$\mu g\,h^{-1}$
ΔR	Radial width of the annulus in which P is not depleted.	mm
d	Agar depth.	mm
$n(\mathbf{R}, t)$	Density of hyphal tips (number per unit volume) at position $\mathbf{R}$ and time t.	mm^{-3}
$\rho(\mathbf{R}, t)$	Hyphal filament density (length per unit volume) at $\mathbf{R}, t$.	$\mu m\,mm^{-3}$
α	Branching rate of hyphal tips.	h^{-1}
β	Anastomosis (fusion) rate of hyphae.	h^{-1}
v_{g}	Average speed of the hyphae that densify the network.	$mm\,h^{-1}$
κ_0	C/P exchange rate (≈3C/P).	dimensionless
$\rho_S = \frac{dS}{dA}$	Spatial hyphal surface area density.	$mm^2\,mm^{-2}$
ξ	Factor accounting for the variance in hyphal radius distribution (≈1).	dimensionless
$\langle r \rangle = a\, v_{\text{wave}} + b$	Empirical affine relation between mean hyphal radius and wave speed; $a = 5.0 \times 10^{-3}\,\mu m\,mm^{-1}\,h^{-1}$, $b = 1.3\,\mu m$.	μm
K_m	Michaelis–Menten half-saturation constant for phosphorus uptake.	$\mu g\,mm^{-3}$
D_P	Diffusion coefficient of phosphate in agar.	$mm^2\,h^{-1}$
J_{max}	Phosphorus-absorption rate per unit hyphal surface area at saturation.	$ng\,mm^{-2}\,h^{-1}$
ϕ_P	Volumetric P absorption rate.	$\mu g\,h^{-1}\,mm^{-3}$

2. Supplementary Methods

A. Plate preparation details.

A.1. Assembly of Mesh Frames. Frames were designed slightly trapezoid to fit next to the central barrier of two-compartment split plates (Greiner Bio-One) with a longer top edge (88 mm) than bottom edge (85.5 mm), and a consistent height (12 mm). They include a central opening (50 x 2 mm) distanced 2 mm from the top edge. This opening connects to the upper edge of the central barrier of the plate, therefore extending the central barrier at the outsides of the plates. The fungus could cross through the opening into the second compartment. To keep the root from crossing over, the opening was covered with nylon mesh.

Acrylic frames were cut using a laser cutter. Nylon mesh (pore size 50 μm, 9 x 71 mm) was attached to the acrylic frame using UV resin in such a manner that the frame opening was fully covered by the mesh and free of resin. The resin was cured with UV light for 3 min, the frames then wrapped in aluminum foil and sterilized at 80°C for 72 hours.

A.2. Two-compartment Split Plate Preparation. In a laminar airflow hood, one compartment of a sterile two-compartment split plate (94 mm diameter, Greiner Bio-One) was filled with 28 mL MSR medium. An autoclaved sheet of cellophane (Hoefer™ TE73, semi-circle with trapezoidal overhang at the straight edge) was placed on top of the solidified medium and the cellophane overhang was folded into the empty second compartment. A custom acrylic frame was inserted into the empty compartment, securing the cellophane overhang between the acrylic frame, central barrier, and the bottom of the plate. To avoid dislocation of cellophane and/or frame, 5 mL 1%P MSR was poured into the second compartment to immobilize the components. The compartment was then filled to a total of 25 mL 1%P MSR.

A.3. Inoculation. In a laminar airflow hood, 2-3 cm of in vitro Ri T-DNA transformed root were transferred to the split plate compartment not covered by cellophane ('root compartment'). A circular plug containing only mycelium and spores was cut from the AMF stock plate (2-6 months). Roots were carefully removed if necessary. The inoculation plug was placed on top of the root, covering not more than half of the root. The plates were sealed with parafilm and stored horizontally and upright in an incubator at 25°C.

A.4. Plate Maintenance. Plates were controlled regularly for fungal and root growth. Any root crossing from the root compartment into the cellophane-cover fungal compartment were pulled back or removed. It usually took 2-4 weeks for the fungal network to start crossing to the fungal compartment and it was kept in the imaging system for 3-6 weeks.

B. Phosphorus Measurements. Measurements and data points are the same as the ones shown in (1). Details are given below.

B.1. Agar and root preparation. We calculated phosphorus concentrations in fungal and root compartments by first removing cellophane from the fungal agar, and then cutting the agar into equal sized pieces (up to 18 pieces). Each agar cube corresponded to a spatial position either away or close to the root. We cut the root compartment agar into two pieces and carefully separated the root from the root agar. We weighed each agar piece and placed it in a Teflon cylinder, and we placed each root in a kraft envelope. We put the Teflon cylinders and envelopes in the oven at 70°C for two days to dry. After drying, we weighed roots and placed them in a Teflon cylinder.

B.2. Digestion. We added 0.5 mL of digestion mixture (HNO3/HCl 4:1) to the Teflon cylinders using a repeating pipette and left the cylinders open for 30 mins to release gases. We then placed the closed cylinders in a destruction oven at 140°C with the temperature limit set to 160°C for 7 hours. We opened the cylinders, added 2 mL of demiwater using a dispenser and transferred the contents of the cylinder to a test tube. We left the test tubes in a fume cupboard for at least one day to release acid fumes and covered them with plastic foil before placing them in the cold room for a week.

B.3. Spectrophotometric determination of phosphate content. The phosphate estimation was based on the formation and reduction of phosphomolybdate. Following the method of (2), we pipetted 150μL of the solution obtained after digestion in test tubes and added 4mL of color reagent. The color reagent was prepared in a 1L water solution with 13.33mL concentrated H2SO4, 1.14g ammonium heptamolybdate, 1.00g ascorbic acid and 0.026g of potassium antimony. The test tubes were left for 30 mins for the color to form. We measured absorbance at 880nm in a spectrophotometer using plastic cuvettes. Because P is known to be bound/adsorbed by soluble aluminium, iron, and manganese at low pH (3), we calculated that $\sim 2\mu$g/mL of P was inaccessible in root and fungal compartments, which we used as our baseline when using equation 3 of Main Text.

C. Timelapse imaging and network segmentation. Imaging, general network segmentation as well as spore segmentation and radius estimation was as described in (1). The rate of false negative was higher for very thin edges especially when focus conditions are not ideal. Such an effect was however estimated to be of small magnitude for most strains. In the case of R. *irregularis*, a higher proportion of very thin edges were not detected. We therefore developed a machine-learning based approach for segmenting the plates from this strain. Such an approach yielded similar results on other strains (Fig.S8) but was better at detecting thinner edges (Fig.S9). Details of the method are given below.

C.1. Label and feature dataset generation. Groundtruth images used for training were obtained from the classic (i.e. non-machine learning) segmentation described in (1), and manually checked by eye for quality. For a set of n=2309 images of R. *irregularis* C2, a segmentation mask (i.e. a binary image where pixels were 1 for hypha, 0 for background) was computed. The raw image was used as input and the segmentation mask as the target output. The full dataset was split into a training set of 1797 images, and a test set of 512 images. In order to mimic typical challenges posed by real data, we employed data augmentation where a random 50% of the training images were blurred, and a random 50% had intrusive background contrast patterns added to them. Although intrusive background contrast patterns were not a major issue for the data considered in this study, they were included in order to train a general purpose AMF segmentation algorithm that is robust to experimental conditions that include such background irregularities.

C.2. Network architecture and training procedure. We used the U-Net CNN architecture (4) as it has achieved impressive results in segmenting biological networks of similar morphology to our fungi (5). U-Net alternates convolutional filters with both max-pool layers, where the image is downsampled by taking only the maximum pixel value in a $N \times N$ region (in our case $N = 2$), and dropout layers. Developed as a method to avoid overfitting, dropout layers temporarily remove a random subset of connections from the network at each training step (6). As such, U-Net first compresses, and then expands the data, resulting in a U shaped architecture. The network also contained skip connections with the aim of preserving large-scale features from the contracting path in the expanding path. Our full model consisted of 31,036,480 trainable parameters.

The network was trained using Stochastic Gradient Descent with Nesterov Momentum (7) according to

$$\theta_{t+1} = \theta_t + v_{t+1}, \quad [1]$$

$$v_{t+1} = \mu v_t - \eta \nabla \mathcal{L}(\theta_t + \mu v_t), \quad [2]$$

where θ_t are the network parameters at timestep t, $\mu = 0.99$ is the momentum coefficient, $\eta = 10^{-4}$ the learning rate, and $\mathcal{L}$ the loss function calculated over a batch. We used a batch size of 1. By a process of trial-and-error experimentation we chose a loss combining the Dice (F1) score and Binary Cross Entropy (BCE),

$$\mathcal{L} = \frac{2\sum_{i=1}^{N} p_i g_i + \epsilon}{\sum_{i=1}^{N} p_i + \sum_{i=1}^{N} g_i + \epsilon} - \frac{1}{N}\sum_{i=1}^{N}\left[g_i \log(p_i) + (1-g_i)\log(1-p_i)\right], \quad [3]$$

where the index i runs over all pixels in an image, p_i is the predicted value for that pixel, g_i the groundtruth, and $\epsilon = 10^{-6}$ is a small parameter for stability. The network was trained for 200 epochs on non-augmented data, and then for a further 150 epochs with augmented data.

C.3. Evaluation. The performance of the network was evaluated in two ways. First, both the classic method and U-Net were used to segment multiple networks of R. *irregularis*. This is a strain where the classic method works well. The total network length was calculated from the resulting pair of segmentations, and compared in Fig.S8. There is a clear positive correlation between the two measurements, from which we conclude U-Net segmentations are in broad agreement with the classic method. The second evaluation method was to visually compare predictions from U-Net and the classic method made on images with high hyphal density. The classic method is known to perform poorly on such examples (e.g. those from R. *irregularis*). A comparison is shown in Fig.S9, demonstrating the significant improvement in hypha detection achieved by U-Net in the case of dense networks.

D. High resolution imaging. As described in (1). The objective used was 50x instead of 100x but the rest of the system was identical.

We built a customized microscope system to acquire high-magnification videos of cytoplasmic flows inside the mycorrhizal hyphae, with the imaging-path optical system identical to that used for low-magnification network imaging (objective, 200mm tube lens and a Basler acA4112-30um CMOS camera, mounted on a Thorlabs KMTS25E/M motorized stage). However, we used a different objective lens (50X Nikon CFI60 TU Plan Epi ELWD). A 1 W red fiber optic LED light source (Product ID: 4165, Adafruit Industries) for illumination through a LED reflector assembled with a Fresnel lens to collect and diffuse the light before the beam reached the sample. The sample stage was a customized X-Y motorized linear stage, with stepper motors (NEMA23 IP20, Servotronix) driven by an Arduino Uno Rev3 micro-controller. With this setup, every video could be related to a specific coordinate in the fungal network.

E. CNN model training and architecture.

E.1. Hyperparameter tuning. We used keras Bayesian Optimization tuner to explore the hyperparameter space. We set the max number of trials to 20 and the number of initial points to 50. The following parameters were adapted.

Convolutional Layers

- Number of Convolutional Layers: Ranges from 1 to 4. The default setting is 2. This determines how many convolutional layers will be added to the model.
- Filters: For each convolutional layer, the number of filters can range from 32 to 256, with a step of 32.
- Kernel Size: The kernel size for each convolutional layer is selectable in a range from 11 to 21 with a step of two. This range was specifically chosen to allow the different hyphal shape to be within the receptive field of the kernel.
- Regularization: Applies L1 regularization on the kernel, activity, and bias with a logarithmic range from 1e-5 to 1e-1 for each, allowing the model to potentially reduce overfitting by penalizing large weights.

Pooling Layers

- Pooling: Can be "MaxPooling" (MP), "AveragePooling" (AP), or "No pool". This choice dictates whether to downsample the feature maps and by which method.

- Pooling Size and Padding: For "MP" or "AP", the pooling layer's size ranges from 1 to 4 with step 1, and padding can be either 'valid' or 'same', impacting the downsampling behavior and the spatial dimensions of the output.

Batch Normalization

- Inclusion: A boolean indicating whether a Batch Normalization layer is added, aimed at stabilizing and accelerating training by normalizing the inputs of activation functions.

Dropout

- Rate: Applied after each dense layer, with a range from 0 to 0.5 in steps of 0.1. This is used to prevent overfitting by randomly setting a fraction of input units to 0 at each update during training.

Dense Layers

- Number of Dense Layers: Ranges from 1 to 4, with a default of 2. This defines how many fully connected layers are added towards the end of the model.
- Units in Dense Layers: For each dense layer, the number of units can range from 8 to 256, with a step of 32. This determines the dimensionality of the layer's output space.

Learning Rate

- Learning Rate: Used by the Adam optimizer, with a logarithmic range from 1e-5 to 1e-1. The learning rate is crucial for controlling the rate at which model weights are updated during training.

Through Bayesian Optimization, these hyperparameters are systematically explored to identify the combination that results in the best performance on the validation set, as measured by the mean absolute error metric.

E.2. Final architecture. Our final model, implemented using the `Keras` framework, is a sequential convolutional neural network designed for processing one-dimensional data. The architecture is summarized as follows:

- The first convolutional layer (`conv_1`) consists of 128 filters with a kernel size of 101 and a stride of 1. It employs the ReLU (Rectified Linear Unit) activation function to introduce non-linearities. This layer is configured with L1 kernel regularization to prevent overfitting by encouraging sparsity in the learned features.
- The second convolutional layer (`conv_2`) also has 128 filters but with a kernel size of 91, maintaining a stride of 1 and using the ReLU activation function. Similar to the first convolutional layer, it includes L1 kernel regularization.
- Batch Normalization: Following the convolutional layers, a Batch Normalization layer is employed to stabilize and accelerate the learning process. This layer normalizes the activations of the previous layer at each batch, maintaining the mean activation close to 0 and the activation standard deviation close to 1.

Dropout Layers:

- The first dropout layer is applied after batch normalization with a dropout rate of 20%. It randomly sets a fraction of input units to 0 at each update during training, which helps in preventing overfitting.
- The second dropout layer follows the first dense layer, with a slightly higher dropout rate of 30%, providing further regularization.
- Flattening Layer: A Flatten layer is used to convert the two-dimensional output of the preceding layers into a one-dimensional array, making it suitable for input into the dense layers. Dense Layers:
- A dense layer with 232 units follows, employing the ReLU activation function. This layer, like the convolutional layers, uses L1 regularization for both kernel and bias. The final output layer has a single unit with a linear activation function, suitable for regression tasks or binary classification.

The total trainable parameters of the model amount to 2,886,097, with an additional 256 non-trainable parameters, leading to a total of 2,886,353 parameters.

E.3. Training.

Final Training Procedure The model was trained using the Adam optimizer with an initial learning rate of 1e-4. The loss function used was mean squared error on the difference between radius squared. We made this choice to avoid underestimation of high radii that represented a small proportion of the dataset. The batch size for training was set at 32, and the model was trained for a maximum of 120 epochs. During training, an EarlyStopping callback was employed to prevent overfitting. This callback monitored the validation mean absolute error, with a patience of 20 epochs and a minimum delta of 1e-3 for the first training phase. After the initial training phase, the learning rate was reduced in two subsequent phases to fine-tune the model: In the second phase, the learning rate was set to 1e-5 with the same batch size and number of epochs. The EarlyStopping callback's patience was reduced to 10 epochs. In the third phase, the learning rate was further reduced to 1e-6, retaining the same batch size, number of epochs, and EarlyStopping configuration as in the second phase. This stepwise reduction in the learning rate is a form of learning rate annealing, which helps in fine-tuning the model parameters and potentially improving the model's performance on the validation set.

E.4. Evaluation.

Independent Test Set Evaluation We reserved an independent test set for final evaluation. The model was trained on the entire training set using the final model and learning rates optimized through hyperparameter tuning. It was then used to make predictions on the test set. The performance was assessed using the same RMSE and R^2 metrics.

Comparison with Linear Regression To benchmark the performance of our neural network model, we compared its results with those from a simple linear regression model. The linear regression model was trained on the same training set and evaluated on the same independent test set. The performance metrics (RMSE and R^2) were calculated in the same manner as for the neural network model.

E.5. Results.

- The null model consisting in taking the average of the training set and using it as a prediction yielded a RMSE of 1.5μm on the resampled test set.
- The linear regression model, used as a baseline, yielded an RMSE of 0.79μm and an R^2 of 0.58 on the resampled test set.
- On the independent resampled test set, the neural network model achieved an RMSE of 0.70 μm and an R^2 of 0.66.
- The distribution of residuals on the training and test sets show that large radius may tend to be underestimated, and smaller radius overestimated. (Fig.S1 B,D).

These results indicate that:

- The neural networks does better than the baseline and the simple linear model
- The RMSE of 0.70 on the test set means that the model predicts a radius value with a 95% confidence interval of +-1.4 μm.
- This error is quite large compared to the typical values of radius. But two points need taking into consideration. When estimating total hyphal surface and volume as it is done in this publication, if radius measurements are (i) independent and (ii) unbiased, error should cancel out. This means that even a large error on radius extraction will translate only in a small relative error on total hyphal surface or biovolume. (i) is justified by the fact that each hyphal radius measurement is taken independently (ii) is not entirely justified given the uneven distribution of residuals, but we can estimate the overall impact of such systematic error.
- The estimated standard deviation of our manual hyphal radius measurement was 0.3μm. This is about 2 times smaller than the RMSE on the test set, which means the neural network is not so far from the maximum precision it could achieve.
- During prediction, multiple transects are used for each edge, and the median of the predicted radius for these transects is used as the edge radius. This tend to mitigate error at the edge level.

Estimating integrated error We estimated the impact of all sources of error (uneven distribution of error along the radius spectrum and quadratic error accumulation) by resampling our radius estimates adding a noise model corresponding to the test set radius dependent RMSE (Fig.S1D). We specifically divided radii in 8 classes from 0 to 8μm. Then for each edge, we calculated its radius as predicted by the model and added an error sample from the residual distribution for that class (assumed gaussian with mean and standard deviation obtained from the test set data). We followed this procedure on 38 mature networks and found that the total biovolume estimate was affected by a few percent (always inferior to 8%, average 5%). We therefore decided to ignore this source of error in subsequent calculations of 95% confidence interval. The biological variability is indeed of larger amplitude than the uncertainty of biovolume extraction due to imperfect radius extraction.

F. Carbon cost and phosphorus absorption calculations from data.

F.1. Computing carbon density and rate of carbon expenditure.

Mass of carbon per unit cell volume We estimate the mass of carbon per unit cell volume M_C as

$$M_C = d_{cell} f_{dry} f_{carbon}$$

where d_{cell} is the mass density of the hyphal cell, f_{dry} is the dry mass fraction of the total (wet) mass, f_{carbon} is the fraction of dry mass accounted for by carbon. We chose $d_{cell} = 1.1\ g/cm^3$ (typical value for cells (8)), $f_{dry} = 21\%$ (typical value for fungi (8)), $f_{carbon} = 50\%$ (typical value for cells (9)). In lieu of known data for spore-specific values for d_{cell}, f_{dry}, and f_{carbon}, we assumed the same values as those for hyphae.

We note that the carbon-estimation parameters d_{cell}, f_{dry}, and f_{carbon} used here should be considered estimates for a generic fungal cell, and hence do not reflect exact values for AM fungi. Yet in lieu of definite evidence for or against these values within the AM fungal literature, we take these generic estimates as our current best approximation. Indeed, previous

study also used very similar estimates for the same parameters for AM fungi (9, 10). We also note that these parameters can in principle vary across hyphae within a given network and over time. Yet given that the formula for C_t (eq. 1) sums over all hyphae and spores of the network, these parameters can be considered network-wide averages that should be quite stable in time during steady travelling-wave growth (11, 12). The limitations of these simplifying assumptions are discussed in further detail in Supplementary discussion A.

Carbon Density To compute the carbon density, we calculated the change in area ΔA and in total network carbon ΔC_t over a time interval Δt. The carbon density was then defined as:

$$\rho_C = \frac{\Delta C_t}{\Delta A}$$

In the case of Fig. 2A we choose $\Delta t = 10h$.

Rate of Carbon Expenditure Rate of Carbon Expenditure was calculated from data with the following formula:

$$\Phi_C = \frac{\Delta C_t}{\Delta t} \times \frac{1}{CUE}$$

F.2. Computing P flux into the network.

P flux in the high P treatment As proposed in previous models of AM fungi P absorption (13):

$$\Phi_P = J_{\max} S(t) \frac{[P]}{[P] + K_m} \quad [4]$$

where $[P]$ represents the concentration of accessible phosphorus in solution in contact with the fungal network. In the regime where $[P]$ is high enough that P transport across the membrane works at saturation (i.e. $[P] \gg K_m$), $J \approx J_{\max}$ and is constant and P depletion proceeds proportionally with the time-integrated surface area $\int_0^{t_m} S(t)dt$ as in Main Text Eq. 4 and computing the P flux Φ_P simplifies from Eq. 4 to Main Text Eq. 5. We don't measure K_m in our experiments but values commonly used in the literature are of the order of $10^{-2} - 10^{-3} \mu g/mL$ which is well below the value $[P]_0 = 1.4 \mu g/mL$ in the high P condition. The fact P_f decays linearly suggests that within the time studied, the concentration of P stays well above K_m. Our use of Main Text Eq. 5 in all cases corresponding to the high P treatment is justified by the fact that all data points correspond to values of the integrated surface area that are well below $10^{10} \mu m^2 h$ and therefore correspond to a regime where this equation is valid according to Fig. 3C. All values in Fig. 4A,B and C were therefore computed using Main Text Eq. 5.

P flux in the low P treatment Computing Φ_P in the low P regime as it is done for Fig. 4E necessitated evaluating Eq.4 at every time point during network growth. To do so, we implemented a three-dimensional explicit finite-difference numerical integration scheme to simulate reaction-diffusion dynamics of phosphorus (P) within a semi-circular petri dish geometry. The computational domain was discretized into a structured grid with dimensions of 30 × 30 × 5 grid points in the x, y, and z directions, respectively, spanning horizontally and to a depth of 8.8 mm, representing the agar layer. Cells had dimensions $\delta x \times \delta y \times \delta z$.

The top layer of cells (i.e. z=0) had a non-zero reaction term that correspond to a volumetric P absorption flux $\phi_P(x, y, t)$ that followed Michaelis–Menten kinetics:

$$\phi_P(x, y, t) = J_{\max} \frac{s(x, y, t)}{\delta z} \frac{[P]}{[P] + K_m}$$

where $s(x, y, t)$ represents the local fungal surface area density (surface area per unit area of agar surface). Values of $s(x, y, t)$ were derived from experimental measurements of fungal surface area across 18 distinct regions of the petri dish, each divided by the area of their respective regions.

The final equation that is being integrated is therefore:

$$\frac{\partial [P]}{\partial t} = D_P \left(\frac{\partial^2 [P]}{\partial x^2} + \frac{\partial^2 [P]}{\partial y^2} + \frac{\partial^2 [P]}{\partial z^2} \right) - \phi_P(x, y, t)$$

Initial phosphorus concentration was uniformly set to $0.5 \mu g P.mL^{-1}$ across the domain. Diffusion was modelled isotropically with a diffusion coefficient $D_P = 1.8 mm^2.h^{-1}$ corresponding to the one estimated for phosphate in Agar gels at 25°C (14).

Zero-flux (Neumann) boundary conditions were enforced laterally at the geometric boundaries of the semi-circular dish and vertically at both the top (surface) and bottom of the agar layer.

Each numerical integration was initiated at a virtual time t_0 before the start of imaging where the total area of the network was estimated to be 0. t_0 was estimated by fitting an affine function to the first 5 measured values of area for each plate and finding the time at which the affine function crossed the y= 0 line. We then extrapolated linearly the measured surface area in each region up until t_0. Such extrapolation was bounded to ensure non-negative values.

Experimental surface area measurements were interpolated linearly in time, decoupling the numerical integration timestep from the experimental imaging frequency.

Numerical stability was ensured by selecting the time step sufficiently small compared to the spatial meshing

$$dt = 0.2\frac{min(\delta x^2, \delta y^2, \delta z^2)}{D_P}$$

Concentration fields were updated iteratively using an explicit Euler scheme, integrating from t_0 to the end of the experiment, capturing transient concentration dynamics and total phosphorus flux. Code for this integration is available in the following repository together with the rest of the replication package.

The total phosphorus flux was computed by integrating reaction terms over all cells.

$$\Phi_P = \sum_i \sum_j \phi_P(x_i, y_j, t)\delta x \delta y \delta z$$

G. Model of network propagation with feedback.

G.1. General Framework. The general framework follows (1). The growing tips are described by their spatial density $n(R,t)$ (with units of number per 3-dimensional volume, e.g. [mm^{-3}]). To account for the hyphal filaments laid down by growing tips, we introduce a variable $\rho(R,t)$, denoting their spatial density (with units of filament length per 3-dimensional volume, e.g. [$\mu\text{m}/\text{mm}^3$]).

Since tip growth deposits hyphal length at a rate v_g, corresponding to the average speed at which hyphae fill space, we set

$$\frac{\partial \rho}{\partial t} = v_\text{g} n$$

Then we describe the local creation and annihilation of tips

$$\frac{\partial n}{\partial t} = \alpha n - \beta n \rho - \nabla \cdot j(n).$$

Where α is the branching rate, β the anastomosis rate and $j(n)$ is a spatial flux that represents the movement of tips across the unit volume.

G.2. Carbon Cost and Network P Absorption from model. We extended the above model so that ρ and n would evolve in a way that would reproduce the observed proportionality between Φ_C and Φ_P. To do so, we first needed to derive those quantities from the model outputs. We start by deriving volumetric quantities representing those fluxes. In the case of the P flux per unit volume ϕ_P, it can be readily obtained from a volumetric version of Main Text Eq. 3.

$$\phi_P(R,t) = J(R,t)2\pi\langle r \rangle \rho(R,t) \quad [5]$$

where $J(R,t)$ is the local rate of P absorption per unit hyphal surface area (see paragraph G.4) and $\langle r \rangle$ is an averaged hyphal radius used for estimating hyphal surface from hyphal length (see paragraph G.5).

At any point in time and space, the amount of carbon used for network growth is proportional to the amount of newly built hyphal volume. Newly built hyphal volume per unit volume is equal to

$$\pi \langle r \rangle_{RMS}^2 \frac{\partial \rho}{\partial t}$$

where $\langle r \rangle_{RMS}$ is an averaged hyphal radius used for estimating hyphal volume from hyphal length (see paragraph G.5 below). The proportionality factor γ_C between this newly built network volume and the amount of carbon used is the product of four parameters: mass density of the cell d_{cell}, the fraction of wet mass accounted for by dry mass f_{dry}, the fraction of dry mass accounted for by carbon f_{carbon}, and the inverse of carbon use efficiency $\frac{1}{CUE}$ so that

$$\gamma_C = \frac{d_{cell} f_{dry} f_{carbon}}{CUE}.$$

We can then define the network carbon building cost per unit volume ϕ_C as

$$\phi_C(R,t) = \gamma_C \pi \langle r \rangle_{RMS}^2 \frac{\partial \rho}{\partial t} \quad [6]$$

Since in our model, the network develops radially around the root, we have cylindrical symmetry and we can compute the total fluxes per unit length root. They are obtained by integrating local volumetric fluxes over space.

$$\Phi^{(\lambda)} = 2\pi \int \phi R dR \quad [7]$$

G.3. Exchange rate driven adaptation. The ratio $\kappa(t) = \frac{\Phi_C^{(\lambda)}(t)}{\Phi_P^{(\lambda)}(t)}$ is the instantaneous exchange rate of Phosphorus in carbon units. We assumed that the network adapts its growth parameter to match an average objective exchange rate κ_0. Specifically, we assumed that the branching rate α is changing over time in order to reach the objective. This is motivated by the fact that branching in fungi is thought to be the consequence of vesicle accumulation at the tips beyond a maximum threshold. In other terms, when the supply of vesicles exceeds their capacity to be incorporated into the existing tip, they accumulate leading to the formation of a new tip (15). Assuming carbon availability is the limiting factor for vesicle production, it is therefore possible that AM adapt to shortage or abundance of carbon by adapting their branching rate. Since the saturation density is a growing function of α, at a fixed propagation speed, Φ_C tends to increase together with α. We therefore assumed that, when the average recent exchange rate $\bar{\kappa}(t) = \frac{\int_{t-\Delta t}^{t} \Phi_C^{(\lambda)}(u)du}{\int_{t-\Delta t}^{t} \Phi_P^{(\lambda)}(u)du}$ overshoots κ_0, α is reduced. Such an integral formulation was chosen to damp oscillatory behavior due to adaptation of branching rate overshooting the objective exchange rate and we chose $\Delta t = 10h$.

We added one last closing equation to the system:

$$\frac{d\alpha}{dt} = -k_2 \left(\bar{\kappa} - \kappa_0\right) \frac{1}{\Delta t} \int_{t-\Delta t}^{t} \Phi_P^{(\lambda)}(u)du.$$

With k_2 a rate parameter. The form is chosen so $\left(\bar{\kappa} - \kappa_0\right) \frac{1}{\Delta t} \int_{t-\Delta t}^{t} \Phi_P^{(\lambda)}(u)du$ has the dimension of a carbon flux, in line with the idea that carbon constrains rather than measurement of the ratio per se leads to the adaptation of α. There are however many forms that the adaptation could take. The main conclusions are however not affected by the specific form of the adaptation as long as it allows the exchange rate to match the set exchange rate on average which is what we could verify with simulations (Fig. 5B).

G.4. Rate of P absorption and P dynamics in the medium.

General case Consistent with the P flux used for estimating P absorption by the network (Main Text, Eq. 7), we use the equation

$$J = J_{\max} \frac{[P]}{[P] + K_m} \tag{8}$$

with K_m the Michaelis-Menten constant for absorption. The dynamics of P-concentration in the medium is then determined by the balance of this flux ϕ_P, which depletes P from the medium, and the diffusive flux $D\nabla^2[P]$, yielding the following partial differential equation (PDE):

$$\frac{\partial [P]}{\partial t} = D_P \nabla^2 [P] - \phi_P \tag{9}$$

where D_P is the diffusion coefficient of P in the medium.

Soil specific P dynamics As explained in (1), P dynamics in agar differ from what can be observed in a real soil. P in soil is in most case reversibly bound to soil particles and only a small fraction is in solution. The equilibrium between the liquid fraction concentration C_L and the solid fraction C_S concentration can be represented with the following relations $\frac{dC_S}{dC_L} = b_p$ where b_p represents the phosphorus buffer power of the soil (13). Within the regime far from saturation of the solid fraction where b_p is a constant, this simplifies to $C_S = b_p C_L$.

The above equations can therefore be adapted to account for this buffering effect. The P concentration experienced by transporters at the hyphal surface is C_L. The expression for J must therefore be adapted to be

$$J = \frac{J_{\max}[P]/b_p}{[P]/b_p + K_m} \tag{10}$$

where $[P] = C_S$ represents the total P bound on soil particles. Then neglecting the diffusion of P on solid surfaces, one can rewrite

$$\frac{\partial [P]}{\partial t} = D_P \nabla^2 ([P]/b_p) - \phi_P \tag{11}$$

This means the diffusion is effectively slowed down by a factor $b_p = 239$ which corresponds to the general consensus for the movement of adsorbed species and specifically Phosphorus in soils (16, 17). Such value can however vary across soil types with sandy soils having values of b_p closer to 1 while the value used here corresponds to clayish soils.

G.5. Accounting for hyphal radius differences.

Dependence of $\langle r \rangle$ on v_{wave} We observed that hyphal extension speed is constrained by radius (Fig. S7A), a relationship consistent with findings in other filamentous fungi (18). Averaged over whole networks, this translated into slower growing networks having smaller $\langle r \rangle$ (Fig. S7B) and $\langle r \rangle_{RMS}$ (Fig. S7C).

This suggests that the hyphal radius $\langle r \rangle$ used for model integration should also vary with speed.

Because even more than five strains would be needed to precisely estimate the functional form to this dependence, we can instead give a generalist functional form $\langle r \rangle = av_{\text{wave}} + b$ with $a = 5.0 \times 10^{-3}h$ and $b = 1.3\mu m$ found by fitting an affine function to the minimum $\langle r \rangle$ as shown in Fig. S7B.

Relationship between $\langle r\rangle_{RMS}$ and $\langle r\rangle$ In order to convert a total length of fungal hyphae into the corresponding network volume we use the length weighted root mean square radius $\langle r\rangle_{RMS}$ where

$$\langle r\rangle_{RMS} = \sqrt{\frac{\sum_i r_i^2 L_i}{\sum_i L_i}}$$

which can be rewritten

$$\langle r\rangle_{RMS} = \sqrt{\left(\langle r\rangle^2 + \frac{\sum_i (r_i - \langle r\rangle)^2 L_i}{\sum_i L_i}\right)}$$

where $\langle r\rangle$ is the length weighted average radius. Setting $\epsilon = \frac{1}{\langle r\rangle^2}\frac{\sum_i (r_i - \langle r\rangle)^2 L_i}{\sum_i L_i}$ which corresponds to the square of the coefficient of variation of the radius distribution we obtain.

$$\langle r\rangle_{RMS} = \langle r\rangle\sqrt{1+\epsilon}$$

At first order, ϵ does not strongly depend on the genotype (see Fig. 1F) and we can therefore set $\xi = 1+\epsilon$ which is considered to be a constant $\xi \approx 1.6$.

H. Numerical simulations. The partial differential equations describing travelling-wave growth of the AM fungal network and P depletion were numerically integrated in the same manner as explained in (1).

H.1. Flux term. We chose $j(n) = -nv_d\hat{\mathbf{R}} + D\nabla n$ with parameter $D = 0.0018$ mm^2/h so that $v_{\mathrm{wave}} = v_d + 2\sqrt{D\alpha} \approx v_d$ and we could more easily vary wave speed.

H.2. Initial condition. Initial conditions were $\rho(R,0) = 0$, $n(R,0) = \frac{q_{max}}{v_g r^2}e^{-\lambda(R-R_0)^2}$ where $q_{max} = 6\times10^{-6}mm^3\,h^{-1}\,mm^{-2}$, $\lambda = 1.2mm^{-1}$ and $R_0 = 7mm$ and $[P](r,0) = [P]_0$. The normalization by v_g was used to ensure that all fungal strategies start with an equal increase in volume density representing a fixed initial carbon investment by the plant $r^2\frac{\partial\rho}{\partial t}(R,0) = r^2 v_{\mathrm{g}} n(R,0) = q_{max}e^{-\lambda(R-R_0)^2}$.

H.3. Other parameters. For all simulations. We chose $\kappa_0 = 3$ corresponding to the value found for AM fungal networks associated with root genotype 1 and $v_{\mathrm{g}} = v_d$. For all simulations, we used $D_P = 3.6mm^2.h^{-1}$ which is the diffusion coefficient of small molecules in water. In Figure 5A, B we choose $k_2 = 0.4mm\,ng^{-1}.h^{-1}$. In Figure 5E, we choose $k_2 = 0.024mm\,\mu g^{-1}.h^{-1}$. The adaptation rate was chosen differently because in Figure 5A,B we wanted to focus on the long term permanent regime travelling wave dynamics and a too large k_2 eventually led to unwanted oscillatory behaviour during integration.

H.4. Integration. In Fig.5A-B: Space was divided in a mesh of 1181 cells from 0 to $252mm$ and time was divided in 900 elements from 0 to $T = 900h$. In Fig.5E, Space was divided in a mesh of 525 cells from 0 to $112mm$ and time was divided in 180 elements from 0 to $T = 180$.

We ran integration using the `dolfin` library for integration of PDEs. Code is available in the following repository together with the rest of the replication package.

H.5. Fig. 5E. For each concentration and each wave-speed we computed the total P transferred to the host plant M_P by integrating Φ_P over time: $P_{tot} = \int_0^T \Phi_P(u)du$. Then at a fixed initial P concentration $[P]_0$, we computed $\bar{P}_{tot}$, the average total transfer of P across all wave-speed sampled. The relative increase in P absorption was $P_{tot}/\bar{P}_{tot}$

We varied v_d over the interval $[150\mu m/h, 320\mu m/h]$ sampling uniformly 20 times. We varied $[P]_0$ over the interval $[2.5\mu g/mL, 25\mu g/mL]$ sampling 20 times.

3. Scaling of Φ_C and Φ_P across travelling wave growth regimes

Because both the carbon expenditure rate Φ_C and phosphorus uptake rate Φ_P increase with network size, it is of interest to ask whether the fixed Φ_C/Φ_P ratio observed throughout our experiments might be a simple consequence of travelling-wave growth of the network. In this section, we therefore derive the expected scaling relationships for Φ_C and Φ_P before, during and after the travelling-wave dynamics become fully established. These scalings clarify that a constant Φ_C/Φ_P ratio throughout network development is by no means a trivial consequence of the observed patterns of growth, and hence suggest the existence of one or more regulation mechanism(s) governing the Φ_C/Φ_P exchange rate.

We identify three regimes of travelling-wave growth to account for dynamics of both hyphal network propagation and P depletion. The first regime corresponds to early times, before the hyphal tip- and filament-density dynamics develop into a travelling wave. The second regime corresponds to intermediate times, where the travelling wavefront of the hyphal network has established, but the concentration of P remains well above saturation ($[P] \gg K_m$) everywhere. In this regime, absorption of P occurs everywhere behind the hyphal network wavefront. The third regime corresponds to late times, and starts upon establishment of a P-depletion front that subsequently advances at the same speed behind the hyphal network wavefront. In this last regime, absorption of P happens only at the periphery of the network within an annulus of fixed width ΔR behind the

hyphal wavefront. In the following sections **A**-**C**, we derive Φ_C and Φ_P scalings for each of these three regimes, together with expressions for the "crossover times" that delineate the regimes. Using our experimental data to calibrate those expressions reveal that the growth data from our experiments correspond mainly to the crossover around t_0 between the early- and intermediate-time regimes. We therefore derive in section **D** an analytical expression for the pareto front corresponding to the expected ρ_S-$v_{\rm wave}$ relationship near t_0, which is used to fit our experimental data in Fig. 5D of the Main Text.

A. Early-time regime: exponential hyphal network growth. In our experiments, the total hyphal length L of the AM fungal network initially exhibits an exponential growth phase (1) where $L(t) = L_0 \exp(\lambda t)$ with L_0 the length of the network at time $t = 0$ and λ an exponential rate constant that we can estimate from experiment ($\lambda \approx 1$ day^{-1}). Differentiating $L(t)$ with respect to time, one obtains $\frac{dL}{dt} = \lambda L$ and hence $\frac{dL}{dt} \propto L$. Throughout this early-time regime, the entire network is exposed to saturating concentrations of phosphorus in the medium (*i.e.* $[P] \gg K_m$ in Eq. 8). Hence, phosphorus absorption Φ_P is proportional to the total hyphal surface area S, which (given cylidrical geometry of hyphae) in turn implies $\Phi_P \propto L$, where the proportionality factor depends on the average hyphal radius. We also have $\Phi_C \propto \frac{dL}{dt}$ since carbon expenditure is proportional to the rate of growth in volume ($\Phi_C \propto \frac{dV}{dt}$), which in turn is (again given cylidrical geometry) proportional to the rate of growth in length. It follows from the proportionality between L and $\frac{dL}{dt}$ that in this regime, $\Phi_C \propto \Phi_P$ where the proportionality factor depends on the exponential rate λ.

B. Intermediate-time regime: Travelling-wave growth prior to P depletion.

B.1. Crossover time t_0 defining the onset of travelling-wave growth. At later times, fungal networks propagate as a travelling wave characterized by wave speed $v_{\rm wave}$ and a hyphal length density ρ_L behind the wave front. In this phase of growth, the total hyphal length L evolves quadratically with time in the half plate:

$$L(t) = \pi\, \rho_L\, v_{\rm wave}^2\, t^2. \tag{12}$$

Now if we let t_0 denote the crossover time delineating exponential and travelling-wave growth, continuity of $L(t)$ requires

$$L_0\, \exp(\lambda\, t_0) = \pi\, \rho_L\, v_{\rm wave}^2\, t_0^2. \tag{13}$$

Our experimental observation of continuity in Φ_C further motivates imposing continuity also in the time derivative of $L(t)$ (because $\Phi_C/\Phi_P \propto (dL/dt)/L$; see above). This yields

$$L_0\, \lambda\, \exp(\lambda\, t_0) = 2\pi\, \rho_L\, v_{\rm wave}^2\, t_0. \tag{14}$$

Dividing Eq. (14) by Eq. (13) and rearranging, we find

$$t_0 = \frac{2}{\lambda}. \tag{15}$$

Plugging the value $\lambda \approx 1$ day^{-1} from our experiments yields $t_0 \approx 2$ days $\approx 50h$.

B.2. General derivation of Φ_C and Φ_P for a hyphal network travelling-wave. For a network growing as a travelling-wave in two dimensions (entirely determined by its saturation density $\rho_{\rm L}$ and its wavespeed $v_{\rm wave}$), we denote $R_{\rm wave}$ its radial extent and ΔR the size of the annulus that is exposed to regions that are not yet depleted in phosphorus. The area of that region is $2\pi R_{\rm wave}\Delta R$ and its rate of P absorption Φ_P is approximately

$$\Phi_P = J_{\rm max}\rho_{\rm L} 2\pi R_{\rm wave}\Delta R\; 2\pi\langle r\rangle \tag{16}$$

Where $J_{\rm max}$ is the Phosphorus-absorption rate per unit hyphal surface area at saturation and $\langle r\rangle$ is the average hyphal radius as defined in SI section 2.G.5. The rate of carbon investment in travelling wave network growth $\Phi_C^{(N)}$, on the other hand, can be estimated to be

$$\Phi_C^{(N)} = \frac{M_C}{CUE} 2\pi R_{\rm wave} v_{\rm wave} \rho_{\rm L} \pi \xi \langle r\rangle^2 \tag{17}$$

where ξ is a constant that accounts for variation in hyphal radius across the network (see SI Section 2.G.5), M_C is the mass of carbon per unit cellular volume and CUE is the carbon use efficiency.

To obtain the total carbon expenditure Φ_C, we yet need to add the rate of carbon investment in spores $\Phi_C^{(S)}$ (which is not directly accounted for in the travelling-wave model). We thus obtain

$$\Phi_C = \Phi_C^{(N)} + \Phi_C^{(S)} = 2\pi \frac{\rho_C}{CUE} R_{\rm wave} v_{\rm wave} + \Phi_C^{(S)} \tag{18}$$

where we have further simplified the expression for $\Phi_C^{(N)}$ by introducing the carbon density $\rho_C \equiv M_C \pi \xi \langle r\rangle^2 \rho_{\rm L}$.

B.3. Scaling of the Φ_C/Φ_P ratio. As long as the whole network is exposed to regions of space where $[P] \gg K_m$, then $\Delta R = R_{\rm wave}$ and $\frac{\Phi_C^{(N)}}{\Phi_P} \sim R_{\rm wave}^{-1}$. So the observation of a constant Φ_C/Φ_P leads to the prediction that there are significant carbon expenditures other than travelling wave growth, such as spore formation, contributing to the measured Φ_C.

C. Late-time regime: Travelling-wave growth with a trailing P-depletion front.

C.1. Crossover time t_1 defining the onset of P-depletion front. At yet later times, P becomes progressively depleted in regions behind the travelling wavefront of hyphal network growth, leading to the establishment of a P-depletion front that trails behind the hyphal growth wavefront. Indeed in our experiments, the hyphal growth wavefront leaves in its wake a network of density ρ_L lying atop the agar gel of depth d, and once a given region reaches the saturation network density ρ_L, it can be estimated that P will be fully depleted after a time

$$\Delta t_1 \approx \frac{[P]_0}{J_{\max} 2\pi \langle r \rangle \rho_{\mathrm{L}}/d} \qquad [19]$$

where $[P]_0$ is the P concentration ahead of the front. For parameter values corresponding to our experiments and the propagation of *R. irregularis* associated with carrot root genotype 1 ($d = 8.8\,\mathrm{mm}$, $[P]_0 = 1.4\,\mu\mathrm{g/mL}$, $J_{\max} = 3.3\,\mathrm{ng\,mm^{-2}\,h^{-1}}$, $\rho_L = 1.5\,\mathrm{mm}^{-1}$, $\langle r \rangle = 2.5\,\mu\mathrm{m}$), this yields $\Delta t_1 \approx 150h$. Consequently, ΔR becomes approximately constant at a crossover time t_1, which we define as

$$t_1 \equiv t_0 + \Delta t_1, \qquad [20]$$

where t_0 is the time of the onset of travelling-wave growth (see section **B.1** above). Given the calibrated values for t_0 and Δt_1 (see above), we thus obtain an estimate $t_1 \approx 8$ days for our experiments.

C.2. Expression for expected constant Φ_C/Φ_P ratio. The constant value of ΔR can be directly estimated from network propagation speed and depletion rate. Indeed, ΔR extends between the current position $R_{\mathrm{wave}}(t) = v_{\mathrm{wave}} t$ of the hyphal growth wavefront and its position $R_{\mathrm{wave}}(t - \Delta t_1)$ at a time Δt_1 ago that marks the edge of the P-depleted zone. This P-depletion front thus moves at the same speed v_{wave} as the hyphal growth wavefront and its spatial extent is thus $\Delta R = v_{\mathrm{wave}} \Delta t_1$. Inserting this expression into Eq.16 yields

$$\Phi_P = 2\pi R_{\mathrm{wave}} v_{\mathrm{wave}} [P]_0 d. \qquad [21]$$

Assuming $\Phi_C^{(S)}$ is close to zero we obtain from equation 18 and 21

$$\frac{\Phi_C}{\Phi_P} = \frac{\rho_C}{CUE[P]_0 d} \qquad [22]$$

which is indeed time independent.

C.3. Iso exchange rate curves (Main Text Eq. 8). In the case where the ratio $\frac{\Phi_C}{\Phi_P}$ is constrained to a fixed value κ_0, we obtain from Eq. 22 by converting the carbon density ρ_C ($= M_C \pi \xi \langle r \rangle^2 \rho_{\mathrm{L}}$) to a surface area density ρ_S ($= 2\pi \langle r \rangle \rho_{\mathrm{L}}$),

$$\rho_S = \kappa_0 \frac{2CUE[P]_0 d}{M_C \xi \langle r \rangle}. \qquad [23]$$

Since $\langle r \rangle = a v_{\mathrm{wave}} + b$ with $a > 0$ (see SI Section 2.G.5), this imposes immediately a trade-off between ρ_S and v_{wave},

$$\rho_S = \kappa_0 \frac{A}{a v_{\mathrm{wave}} + b} \qquad [24]$$

where $A = \frac{2CUE[P]_0 d}{M_C \xi}$. Although our data does not correspond to this permanent regime, the above derivation helps to illustrate how the trade-off between ρ_S and v_{wave} emerges, as well as the dependence of saturation density of the hyphal network on initial phosphorus concentration. The trade-off defined by Eq. 24 is illustrated by the plotted iso-lines in Fig. 5C where parameters have been fitted to the same data as the one shown in Fig. 5D. After computing the Pareto front for each root genotype, we fit Eq. 24 to both fronts using $\kappa_0 = 3$ for genotype 1 and $\kappa_0 = 4$ for genotype 2 using `curve_fit` of `scipy` package of Python. This yields a value for A which is then used to plot the curves in Fig. 5C of Main Text using Eq. 24. In order to understand the variation as it is observed in our experiment (Fig. 5D), we use a different framework (see Fitting of Pareto front section below).

D. Fitting of Pareto front (Fig. 5D).

The values for the crossover times $t_0 \approx 50$ h and $t_1 \approx 200$ h estimated in sections B and C above, respectively, imply that the growth dynamics observed in our experiments ($0 < t < 100$ h) are centered about t_0 at the transition between the early- and intermediate-time regimes. For the Pareto-front analysis of the experimental data in Fig. 5D, we therefore need to derive the iso exchange rate curve expressing the relationship between ρ_S and v_{wave} at constant exchange rate κ_0 that is valid around the crossover time t_0 between the exponential and travelling-wave growth phases.

In section **A** above, we established that a constant Φ_C/Φ_P ratio is expected from exponential growth dynamics. With similar reasoning, it can be shown that the exponential growth rate is proportional to a fixed exchange rate. Indeed, for a growing network with fixed average hyphal radius $\langle r \rangle$, assuming $[P] >> K_m$ and no carbon investment into spore biomass (which we experimentally verified at early times; see SI Fig. S4) and a fixed exchange rate κ_0 ($\equiv \Phi_C/\Phi_P$), we can write,

$$\frac{M_C}{CUE} \pi \xi \langle r \rangle^2 \frac{dL}{dt} = \kappa_0 \, 2\pi \langle r \rangle \, J_{\max} \, L, \qquad [25]$$

which can be solved to yield exponential dynamics of the form:

$$L(t) = L_0 \exp\left(\frac{2CUE\kappa_0 J_{\max}}{M_C \xi \langle r \rangle} t\right) = L_0 \exp(\lambda t), \quad [26]$$

where L_0 is the initial length, and the growth rate $\lambda \equiv \frac{2CUEJ_{\max}}{M_C\xi\langle r\rangle}\kappa_0$ is indeed proportional to κ_0.

Using this expression for λ to evaluate t_0 in Eq. 15 and plugging into Eq. (13), we obtain

$$L_0 e^2 = \pi \rho_L v_{\text{wave}}^2 \left(\frac{M_C \xi \langle r \rangle}{CUE\kappa_0 J_{\max}}\right)^2,$$

which rearranges to

$$\rho_L = \frac{L_0\, e^2}{\pi\, v_{\text{wave}}^2} \left(\frac{CUE\kappa_0 J_{\max}}{M_C \xi \langle r \rangle}\right)^2. \quad [27]$$

If we choose L_0 so networks of different average radii always start with the same volume $V_0 = \pi\xi\langle r\rangle^2 L_0$ at $t = 0$ (corresponding to a given initial carbon expenditure) and define the constant $\epsilon \equiv \frac{e^2 CUE^2 J_{\max}^2}{\pi^2 \xi^3 M_C^2}$, we can express this compactly as:

$$\rho_L = \epsilon \frac{V_0}{\langle r \rangle^4} \left(\frac{\kappa_0}{v_{\text{wave}}}\right)^2, \quad [28]$$

from which we obtain an expression for the Pareto front relating the achievable surface area density ρ_S to network range expansion speed v_{wave} when all received carbon from the plant is invested in network growth (i.e. no spore investment; $\Phi_C^{(S)} = 0$ in Eq. 18),

$$\rho_S = 2\pi\epsilon \frac{V_0}{\langle r \rangle^3} \left(\frac{\kappa_0}{v_{\text{wave}}}\right)^2. \quad [29]$$

Where $\langle r\rangle = av_{\text{wave}} + b$ (see SI Section 2.G.5). So Eq. 29 can be rewritten:

$$\rho_S = 2\pi\epsilon V_0 \frac{\kappa_0^2}{f(v_{\text{wave}})}. \quad [30]$$

where $f(v_{\text{wave}}) = (av_{\text{wave}} + b)^3 v_{\text{wave}}^2$. V_0 is an arbitrary parameter that can be adjusted. After computing the Pareto front for each root genotype, we fit Eq. 29 to both fronts using $\kappa_0 = 3$ for genotype 1 and $\kappa_0 = 4$ for genotype 2 using `curve_fit` of `scipy` package of Python. This yields a value for V_0 which is then used to plot the curves in Fig. 5D of Main Text using Eq. 29.

4. Supplementary discussion

A. Limitations of carbon estimates.

- **Variations in Cell Wall Thickness and Lipid Density:** The estimate of carbon flux given in the Methods does not consider variations in cell wall thickness or lipid density inside the cells which can vary from hypha to hypha (19, 20). Further refinements can include such considerations, but we expect the carbon ratio of cells to be close to the one used in this study.

- **Uncertainty in CUE value:** It is important to note that CUE reported in the literature can encompass variable definitions and measurement methods. We didn't find any specific estimates for AM Fungi that would match our criteria. In nutrient poor Boreal forests, CUE of ectomycorrhizal (EM) fungi was found to vary between 3 and 15% (21). We however expect AM fungi CUE to be significantly higher since, on the contrary to EM fungi, they invest no carbon in substrate decomposition. While our estimate of CUE for AM fungi is uncertain, it does not affect the main conclusion of the paper since a different CUE, if it is not strain specific, will not break the observed proportionality between P absorption by AMF and C use but simply change the proportionality factor.

- **Maintenance Cost, Carbon Recycling, and Spores:** At the timescale considered (4-5 days) we considered that some more complex carbon dynamics could be neglected. All the different possible carbon costs for a growing fungal network are nicely summarized and quantified in (22).

  ⋆ First, we neglected the carbon cost associated with cell maintenance. This cost, although of smaller magnitude, is proportional to total network length, while the cost associated with growth is proportional to the derivative of the total length. It has been estimated for fungi that the time over which the metabolic cost of maintaining a living volume is equal to the number of joules embodied in that volume was about 30 days for fungi (22). At the timescale considered it is therefore valid to consider the maintenance cost negligible.

- ⋆ We also neglected the cost associated with transport of resources within the network. This was motivated by the following calculation. Assuming all the cytoplasm is moving at $v_0 = 3\mu m.s^{-1}$ in $L = 1m$ of network of radius $r = 3\mu m$. The hydraulic resistance associated with the network is $R_h = \frac{8\mu L}{\pi r^4}$ where $\mu = 1g.m^{-1}.s^{-1}$ is the viscosity of the cytoplasm and the flux through the pipe is $Q \approx \pi R^2 v_0$. The energy dissipation power is then of the order of $P_{transport} = R_h Q^2 = 8\pi\mu L v_0^2$ which does not depend on R. We can therefore evaluate that $P_{transport} = 226 \times 10^{-15} W \approx 0.2 \times 10^{-6} \mu W$. In comparison, we estimate that the growing networks can consume $\Phi_C \approx 0.2\mu g/h$ of carbon (see Fig. 2B). This carbon is in the form of lipids/palmitic acid which energy density is about $\epsilon = 37MJ/kg$. The Power requirement for growth can therefore be estimated to $P_{growth} = \epsilon\Phi_C \approx 2\mu W$. At first approximation, the energetic cost of transport therefore seems negligible compared to the energetic cost of growth.
- ⋆ Fungi can also recycle cell material. To generate the dataset used for machine learning, we sampled multiple positions in the fungal network over several days. At the timescale considered, we didn't observe hyphal cytoplasm retracting except for thin Branched Absorbing Structures (BAS) around the end of that timescale. It was previously estimated that full BAS lifecycle consisted in 7 days for formation followed by 5 weeks until full retraction (19). We observe a faster BAS lifecycle in our experiments (some hyphae starting to retract after just 5 days). Interestingly the timescale of BAS retraction seems to coincide with the one of P depletion. We however decided not to include this effect in our calculation since (i) BAS constitute 30% of total hyphal length (see (1)) but are thinner than the rest of the hyphae. We expect they constitute no more than 10-15% of total hyphal volume (ii) While some BAS could indeed start to retract over a similar timescale as the one of our experiments, it was clearly not the case for most of them, so we expect the overall amount of recycling to be negligible at the timescales considered.
- ⋆ Finally, spores may well have a higher carbon mass per unit volume than hyphae, given that they store lipids as fuel for germination. However, they also have thicker cell walls, which might offset some or all of the difference in carbon cost per unit volume. We therefore used the same conversion factor M_C to compute total network carbon from the total fungal biovolume including all hyphae and spores. At the timescales considered, spores only constitute a small fraction of total volume. We therefore expect any inaccuracies due to this simpliflying approximation to be negligible.

- **Carbon investment flux in the root compartment:**
  - ⋆ Our imaging extra radical hyphal growth does not extend to the root compartment. It is however likely that, because phosphorus is already depleted in that compartment (see Methods), hyphal growth is also negligible.
  - ⋆ Extra radical hyphal growth is generally accompanied by intra radical hyphal growth that also consumes carbon. However, at the timescales considered (first 10 days), intra-radical growth can be considered negligible in length and probably even more in volume (1).
- **Geometrical constraints:** Because our experiments are done in a finite space (half petri dish), it is expected that geometrical constraints could play a role in the allocation of carbon. An extreme case is the fact that when the whole plate is covered with fungal hyphae, no more growth happens and Φ_C goes to zero. In order to mitigate these effects In the case of Fig. 2 and 4 we:
  - ⋆ Never included data points more than a 100 hour after the network area has reached $200mm^2$
  - ⋆ Never include data points after a maximum time set as the one corresponding to the maximum observed rate of biovolume growth.

B. Limitations of phosphorus estimates.

- **BAS and Runner Hyphae:** Estimates of structure specific expression of P absorption genes showed higher relative expression of P-transporters in BAS (23). The normalisation is however done on volume and not surface, and it is unclear from these results whether the transporter density on the surface of BAS hyphae should be considered higher. We therefore decided not to make any distinction between different hyphal structures beyond their radii.
- **Hyphal Septation:** As explained above, at the timescales considered, we decided not to consider the possibility that some subpart of the hyphal network could retract and septate and therefore not contribute to P absorption. In addition, it is likely that BAS retraction coincides with P depletion and we already account for the fact that hyphae in P depleted region do not contribute to the total absorption by the network.
- **Root compartment absorption:** We only observe hyphae present in the fungal compartment (upper half). However, hyphae that could be absorbing phosphorus can also be present in the root compartment. We do not account for those in our estimates. This is however justified by the fact that at the moment where hyphae are crossing in the fungal compartment, phosphorus is generally already depleted in the root compartment (see Fig.3 and SI section 2.B).

C. Analytical considerations on the optimal wave speed. The results shown in Fig. 5E can be understood from an analytical perspective.

C.1. Low P, Permanent regime. In the permanent regime, letting $R = v_{\text{wave}} t$ within the expression for Φ_P derived above, we have

$$\Phi_P(t) = \pi v_{\text{wave}}^2 [P]_0 t,$$

which means the fastest expanding strains (with high v_{wave}) will always provide more P. This is understandable since the permanent regime corresponds to a regime where escaping the P depletion zone is essential.

C.2. High P, Exponential regime. At high P ($[P] >> K_m$), for a growing network with fixed average hyphal radius $\langle r \rangle$, equating the carbon expenditure on network growth Φ_C with the phosphorus flux Φ_P scaled by a fixed exchange rate κ_0 yields

$$\frac{M_C}{CUE} \pi \xi \langle r \rangle^2 \frac{dL}{dt} = \kappa_0 \, J_{\max} \, 2\pi \langle r \rangle L, \quad [31]$$

which is a differential equation describing dynamics of the total network length L. This equation has the solution

$$L(t) = L_0 \, \exp\left(\frac{2CUE\kappa_0 J_{\max}}{M_C \xi \langle r \rangle} \, t \right), \quad [32]$$

where L_0 is the network length at time $t = 0$. If we choose L_0 so networks of different average hyphal radii always start with an equal volume V_0 at $t = 0$ we have

$$L(t) = \frac{V_0}{\pi \xi \langle r \rangle^2} \, \exp\left(\frac{2CUE\kappa_0 J_{\max}}{M_C \xi \langle r \rangle} \, t \right), \quad [33]$$

Integrated over time we obtain

$$P_{tot} = \int_0^T \Phi_P(u) du = \frac{M_C}{CUE\kappa_0} V_0 \, \exp\left(\frac{2CUE\kappa_0 J_{\max}}{M_C \xi \langle r \rangle} \, T \right),$$

which is a decreasing function of $\langle r \rangle$ meaning that strains with smaller radii (i.e. slower wave speed) will always achieve higher P-uptake performance in this regime.

C.3. Optimal propagation in the non P depleted travelling wave regime. Before reaching the permanent regime of travelling-wave growth with P depletion, there is an intermediate regime in which travelling-wave growth is established but the P-depletion front has yet to fully establish. In this regime, when P uptake remains saturated ($[P] >> K_m$), we have

$$\Phi_P(t) = J_{\max} \rho_{\text{L}} \pi v_{\text{wave}}^2 t^2 \; 2\pi \langle r \rangle.$$

Using Eq. 28, we obtain

$$\Phi_P(t) = \frac{1}{\langle r \rangle^3} J_{\max} A \left(\frac{\kappa_0 J_{\max}}{M_C} \right)^2 2t^2 \; \pi^2.$$

Where $A = \frac{\epsilon V_0}{\xi^3}$ This means that strategies with smaller radii will provide higher Φ_P. Given the linear relationship between v_{wave} and $\langle r \rangle$, this expression clarifies why slower growing strains can achieve better P transfer performance at low $[P]_0$ (Fig. 5E of Main Text).

665 ## 5. Supplementary table

Table S2. Parameters used across the manuscript

Parameter	Description	Unit	Value	Used in Fig.	origin
d_{cell}	Characteristic cell mass density	$g.cm^{-3}$	1.1	Fig. 2, Fig. 4 and Fig.5	(8)
f_{dry}	Fraction of dry biomass	.	0.21	Fig. 2, Fig. 4 and Fig.5	(8)
f_{carbon}	Fraction of carbon in dry biomass	.	0.5	Fig. 2, Fig. 4 and Fig.5	(9)
J_{max}	Maximum uptake rate per unit surface area	ng P $mm^{-2}\,h^{-1}$	3.3	Fig. 2, Fig. 4 and Fig.5	measured
K_m	Michaelis-Menten half-saturation constant	μg P mm^{-3}	3.1×10^{-5}	Fig. 4 and Fig.5	(13)
CUE	Carbon use efficiency during growth	.	0.5	Fig. 2, Fig. 4, Fig. 5	(24)
M_C	mass of carbon per unit cell volume	μg C mm^{-3}	115	Fig. 2 and Fig. 4	$= d_{\text{cell}} f_{\text{dry}} f_{\text{carbon}}$
γ_C	Conversion factor between hyphal length biovolume growth rate and carbon expenditure rate	μg C mm^{-3}	231	Fig. 5	$= \frac{d_{\text{cell}} f_{\text{dry}} f_{\text{carbon}}}{CUE}$
D_P	Diffusion coefficient of phosphate ions	$mm^2.h^{-1}$	1.8 in Fig. 4E, 3.6 otherwise	Fig. 4, Fig. 5	(14)

6. Supplementary figures

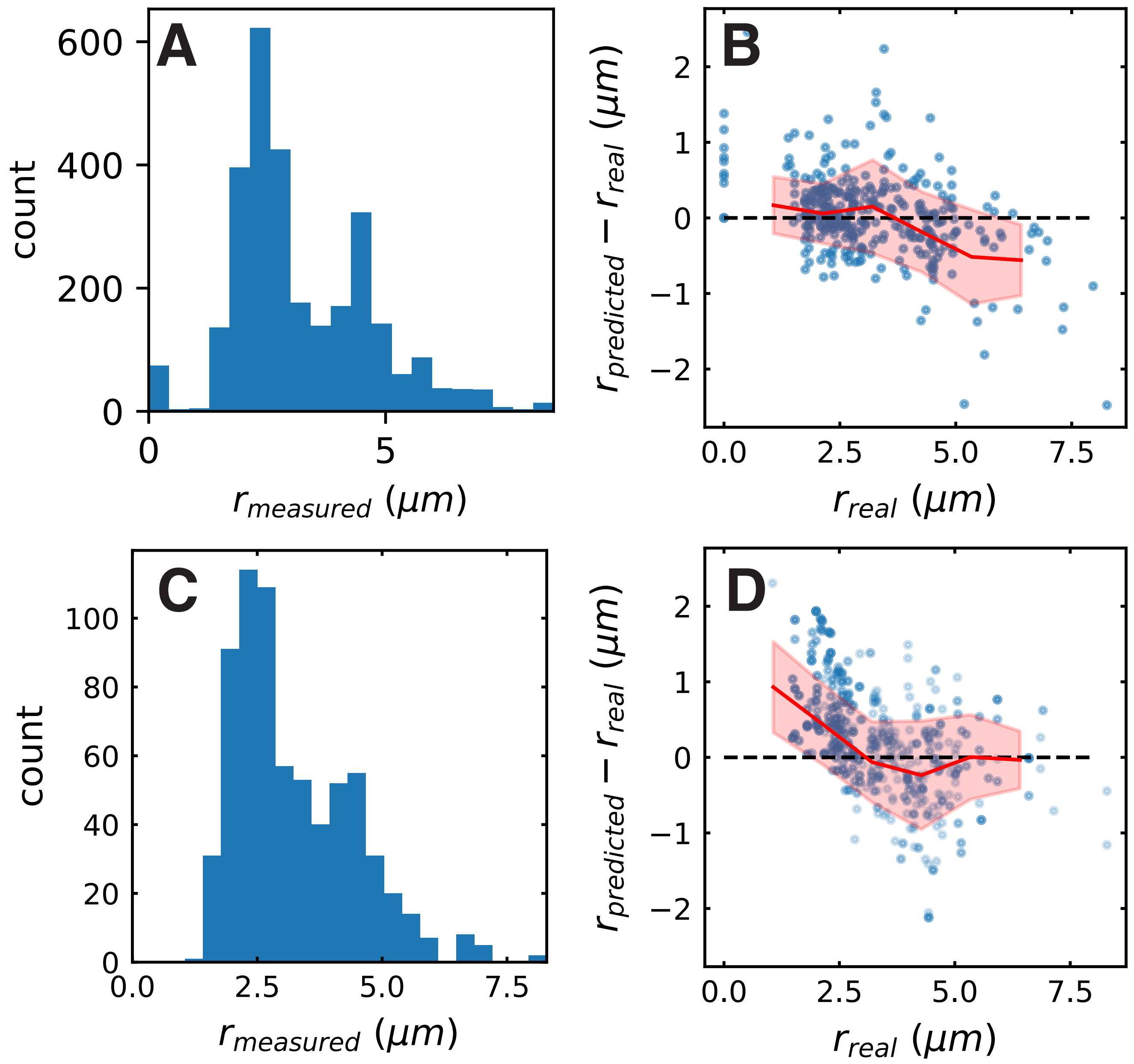

Fig. S1. Training and test set distribution and model performance on validation and test sets. (**A**) Training + validation set distribution. (**B**) Residual of the model prediction on the validation set. (**C**) Test set distribution after resampling. (**D**) Residuals of the model prediction on the resampled test set. For (**B**) and (**D**), blue points correspond to individual predictions from the set. Radii were separated in 8 classes from 0 to 8μm. For each class, the mean residual and the standard deviation were computed. Red line links the mean residual for each class and red shade shows the 95% C.I. for each class (mean ± 2 × s.e.m.).

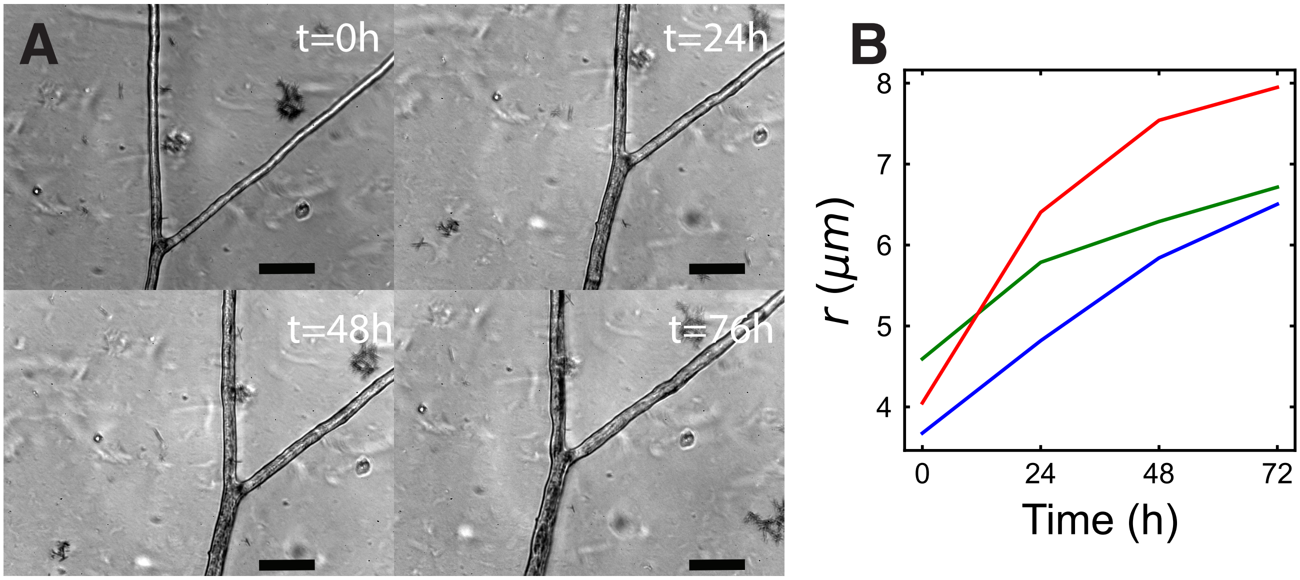

Fig. S2. High resolution imaging shows hyphal widening over time. (A) High resolution images of the same Y-shaped intersection over 4 days. Scale bar is 40 μm. **(B)** Average of measured radius of the three edges of the intersection over time (Bottom : red, upper-left: green, upper-right: blue)

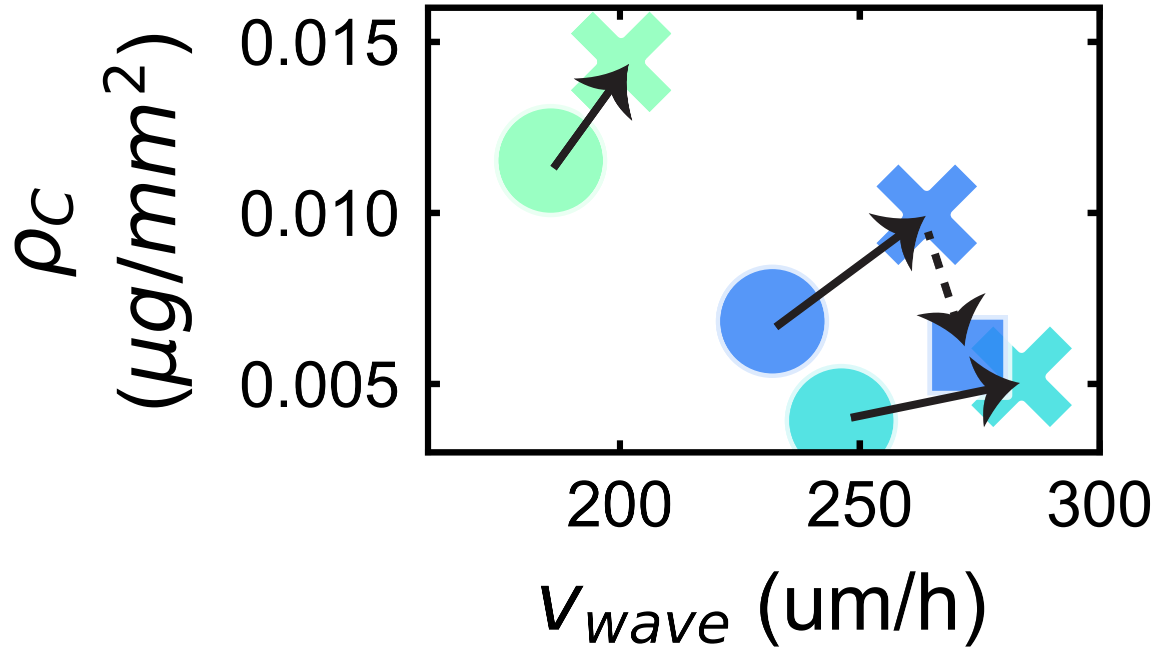

Fig. S3. Effect of changing host genotype, and environmental P concentration, on travelling-wave parameters. Travelling wave observables ρ_C and v_{wave} represent the instantaneous hyphal carbon density and the instantaneous wave speed, respectively. Symbols represent the median of those values for each strain. Dark blue corresponds to *R. irregularis* strain C2, cyan to strain A5 and green to *R. aggregatus*. Circles correspond to carrot root genotype 1 and high P environment in the fungal compartment, crosses correspond to carrot root genotype 2 and high P environment, square correspond to carrot root genotype 2 and low P environment. Arrows are guide for the eye. Continuous arrow corresponds to a change from genotype 1 to genotype 2, dashed arrow corresponds to a change from high to low P environment.

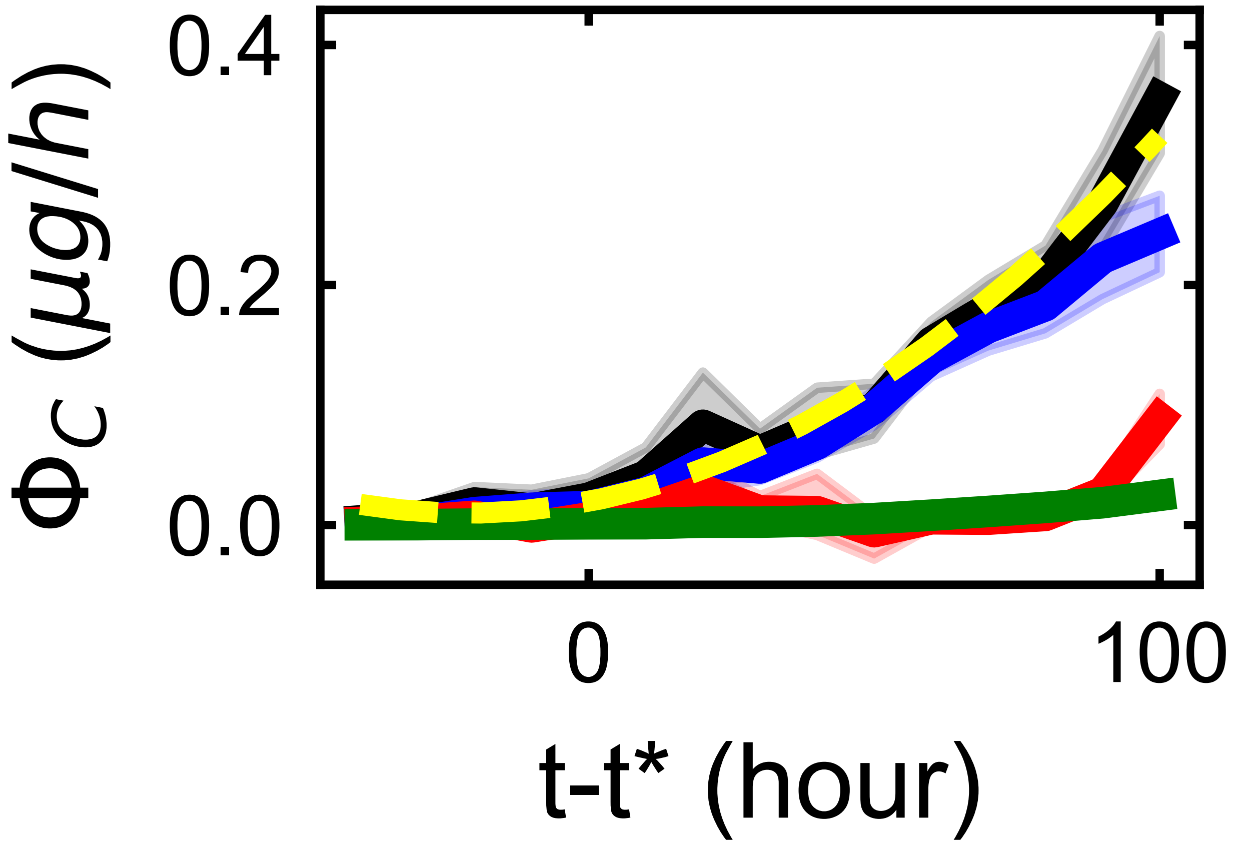

Fig. S4. Comparison of different contributions to carbon expenditure by the network Carbon spent by the growing network in hyphal structures (blue), spores (red), respiration (green) and total (black) and per hour as a function of time. Thick line and shade represent average and 95% confidence interval of all replicates' dynamics. Average and 95% confidence interval (mean $\pm 2\times$ s.e.m.) are computed over 10-hour time intervals. Maintenance costs were estimated by multiplying total fungal biomass by a factor $k = 4.5 \times 10^{-4} h^{-1}$. k is obtained by dividing an average mean mass-specific metabolic rate of $5 \times 10^{-9} W \cdot \mu g^{-1}$ (25) by an energy per unit mass carbon oxidized of $40 \times 10^{-3} J \cdot \mu g^{-1}$ corresponding to typical carbohydrate complete oxidation (26). Yellow dashed line correspond to a quadratic fit of the total carbon expenditure. Such quadratic growth is expected in the regime where Φ_P also grows quadratically and is proportional with Φ_C

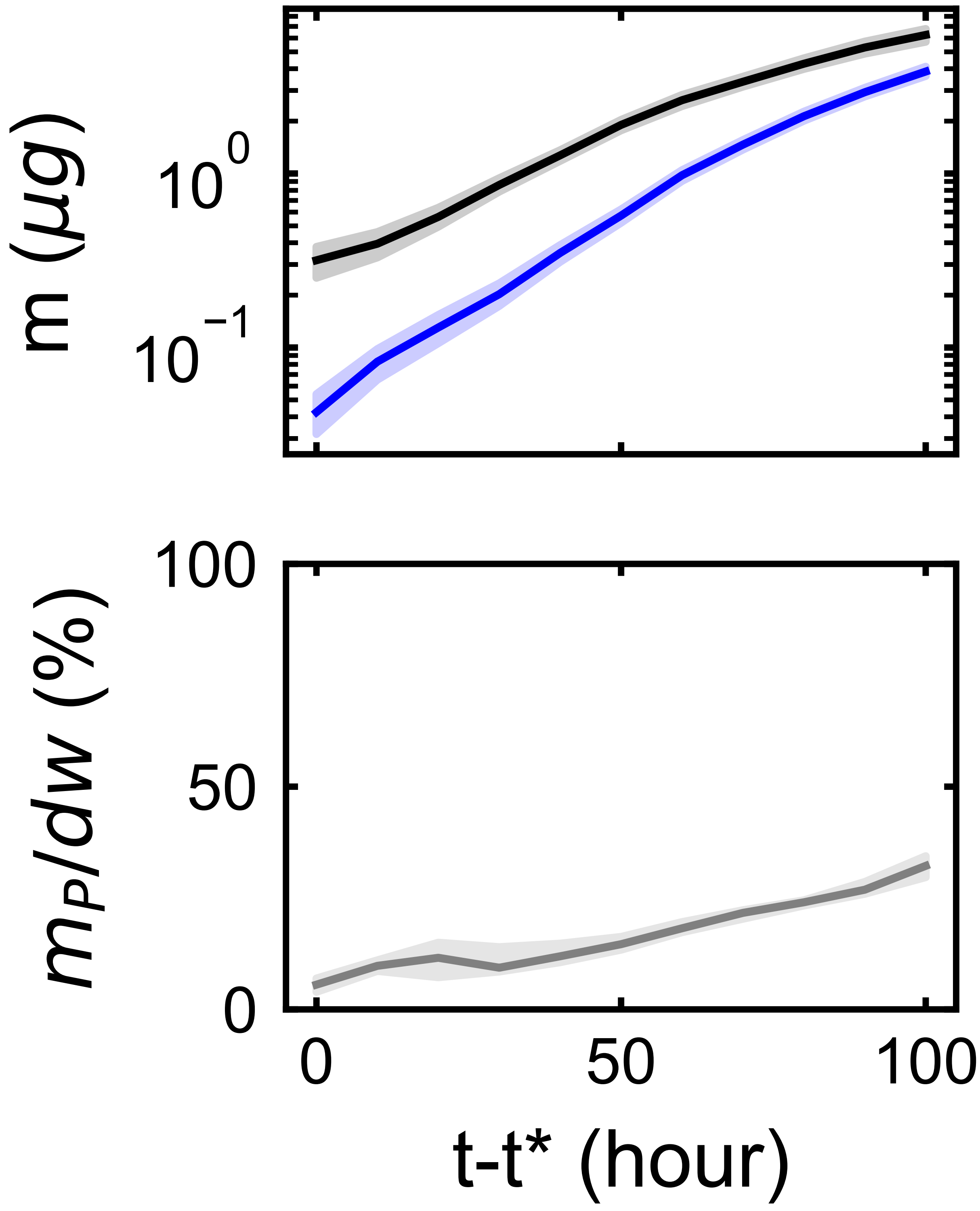

Fig. S5. Mass stoichiometry of fungal hyphae under the hypothesis of no transfer to the plant. Estimates of total carbon mass (black), total absorbed phosphorus mass (blue) and ratio of the two (grey) as a function of time. Absorbed phosphorus mass is estimated from the measured temporal dynamic of network surface area. Thick lines correspond to average of all replicates over 10 hour time intervals, shades correspond to 95% C.I. over the same interval (mean $\pm 2\times$ s.e.m.).

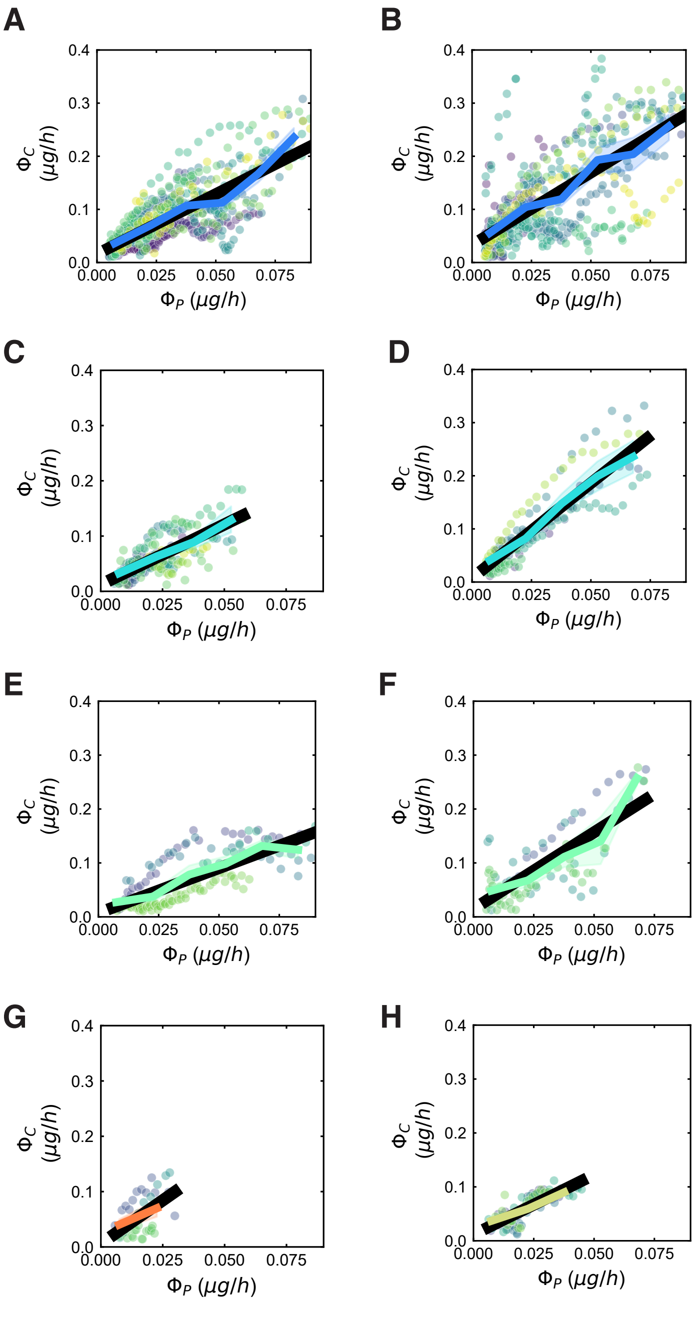

Fig. S6. Proportionality of C expenditure and P supply for each plant-fungus strain combination. Carbon expenditure rate (Φ_C) as a function of Phosphorus supply rate (Φ_P). **(A) ,(C),(E),(G),(H)** correspond to genotype 1 carrot root. **(B) ,(D),(F)** correspond to genotype 2 carrot root. **(A), (B)** correspond to *R. irregularis* C2. **(C), (D)** correspond to *R. irregularis* A5. **(E), (F)** correspond to *R. aggregatus*. **(G)** corresponds to *R. irregularis* C3. **(H)** corresponds to *R. clarus*. Each point corresponds to a measurement of Φ_C and Φ_P at one timestep for one replicate, each replicate is shown in a different color. colored line shade corresponds to binned average 95% C.I. over regular Φ_P intervals. Black line corresponds to linear fit over the blue points. For *R. irregularis* A5 (cyan; $n_{\text{genotype 1}} = 8$, $n_{\text{genotype 2}} = 6$),C2 (blue; $n_{\text{genotype 1}} = 19$,$n_{\text{genotype 2}} = 11$), *R. aggregatus* (green, $n = 4$), *R. clarus* (yellow, $n = 4$) associated with genotype 2 (dashed line) and genotype 1 (full line).

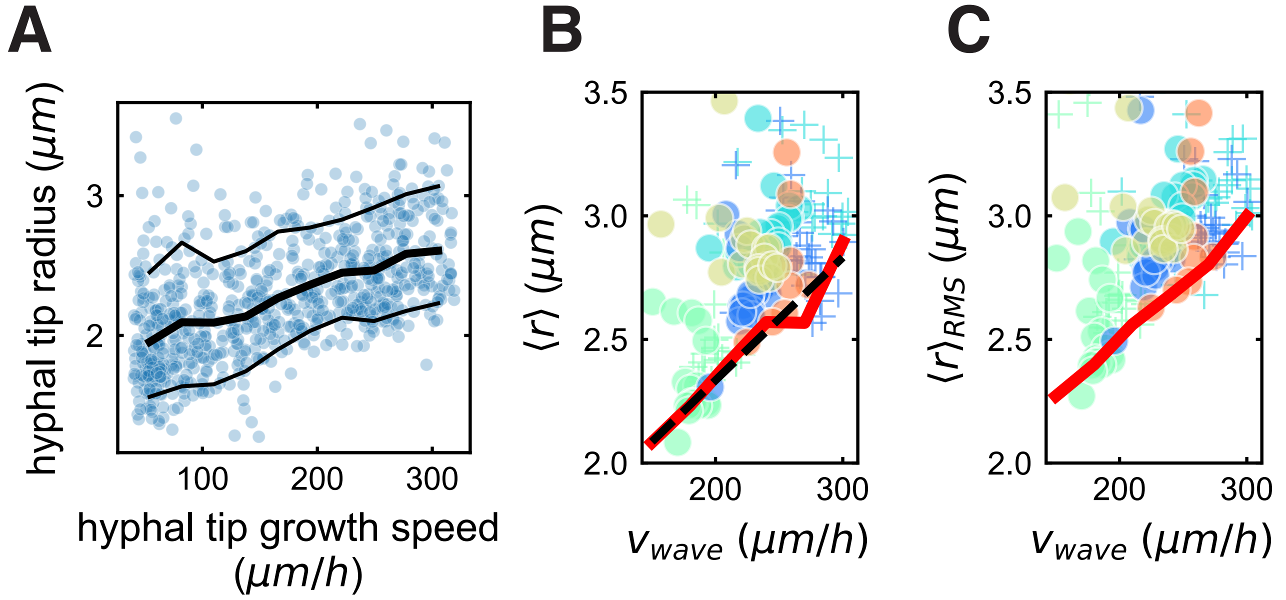

Fig. S7. Fast expansion is constrained by hyphal width. (A) Individual hyphal growth speed is constrained by tip radius. Each blue point corresponds to one growth movement of a hypha between two timesteps. Middle thick black line links averages. Upper and lower black lines link respectively the 90th and 10th percentiles. Statistics are computed in 10 bins over the range of hyphal tip growth speeds. **(B)** Each small circle corresponds to an independent median of mean length weighted radius over 10 hours for all plates of the same strain (n = 7-11). Mean length weighted radius for a given plate at a given time is computed by dividing the newly created total surface area by 2π times the newly created length. Red line links the minimum over bins of size 30$\mu m/h$. Black line correspond to the linear fit of the points that constitute the red line. **(C)** Each small circle corresponds to an independent median of length weighted root mean squared radius over 10 hours for all plates of the same strain (n = 7-11). Length weighted root mean squared radius for a given plate at a given time is computed by dividing the newly created total biovolume by π times the newly created length and taking the square root. Red line links the minimum over bins of size 30$\mu m/h$. In **(B)** and **(C)**, color correspondence and number of replicates are the following: *R. irregularis* A5 (cyan, n=8), C2 (blue, n =19) and C3 (red, n=2), *R. aggregatus* (green, n=4), *R. clarus* (yellow, n= 4)

.

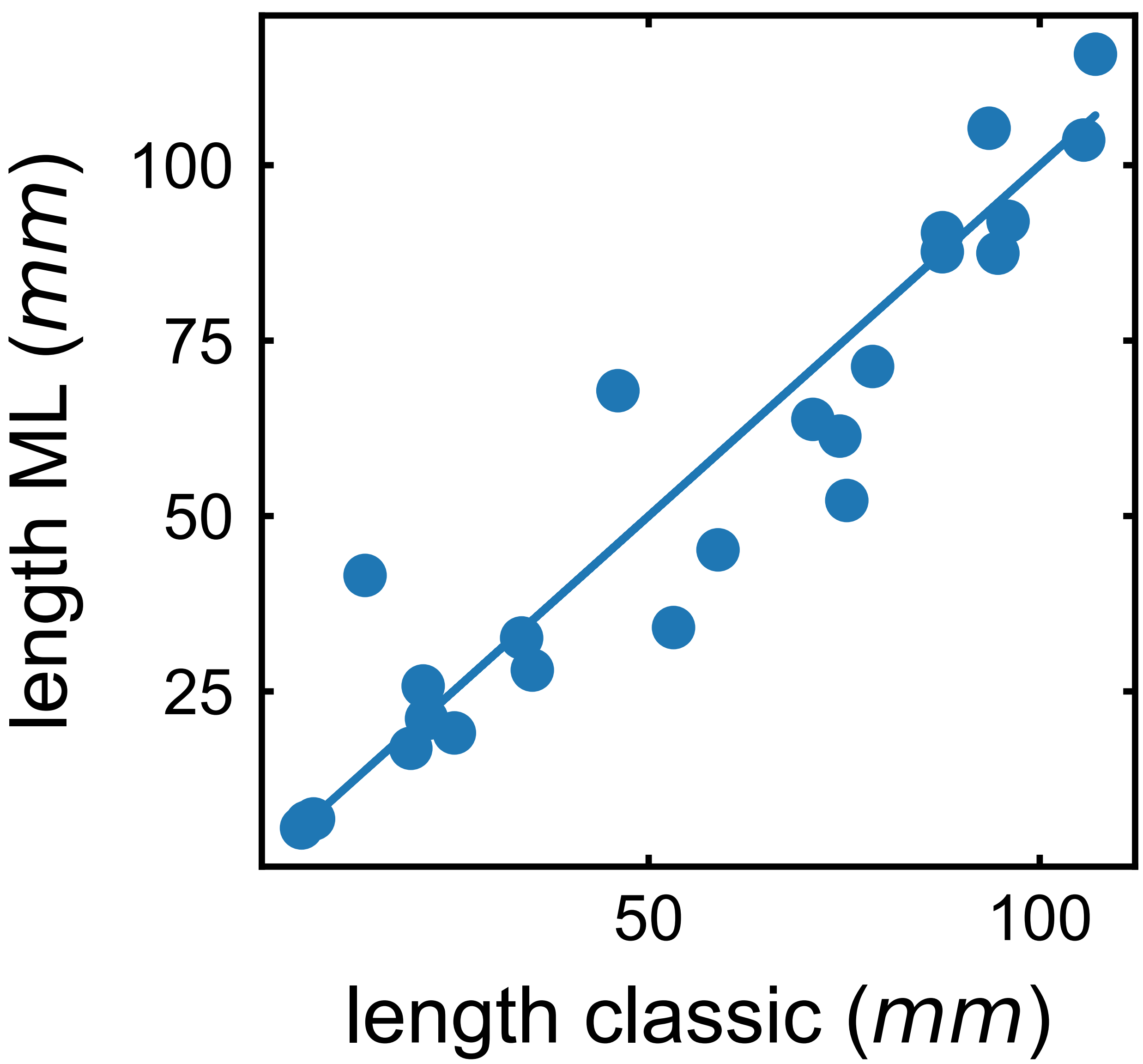

Fig. S8. Two segmentation methods yield similar results on R. *irregularis*. Each point is obtained from comparing total length obtained through segmentation of a single tile either with the classic algorithm or with the machine learning based one. Blue line is a 1:1 line.

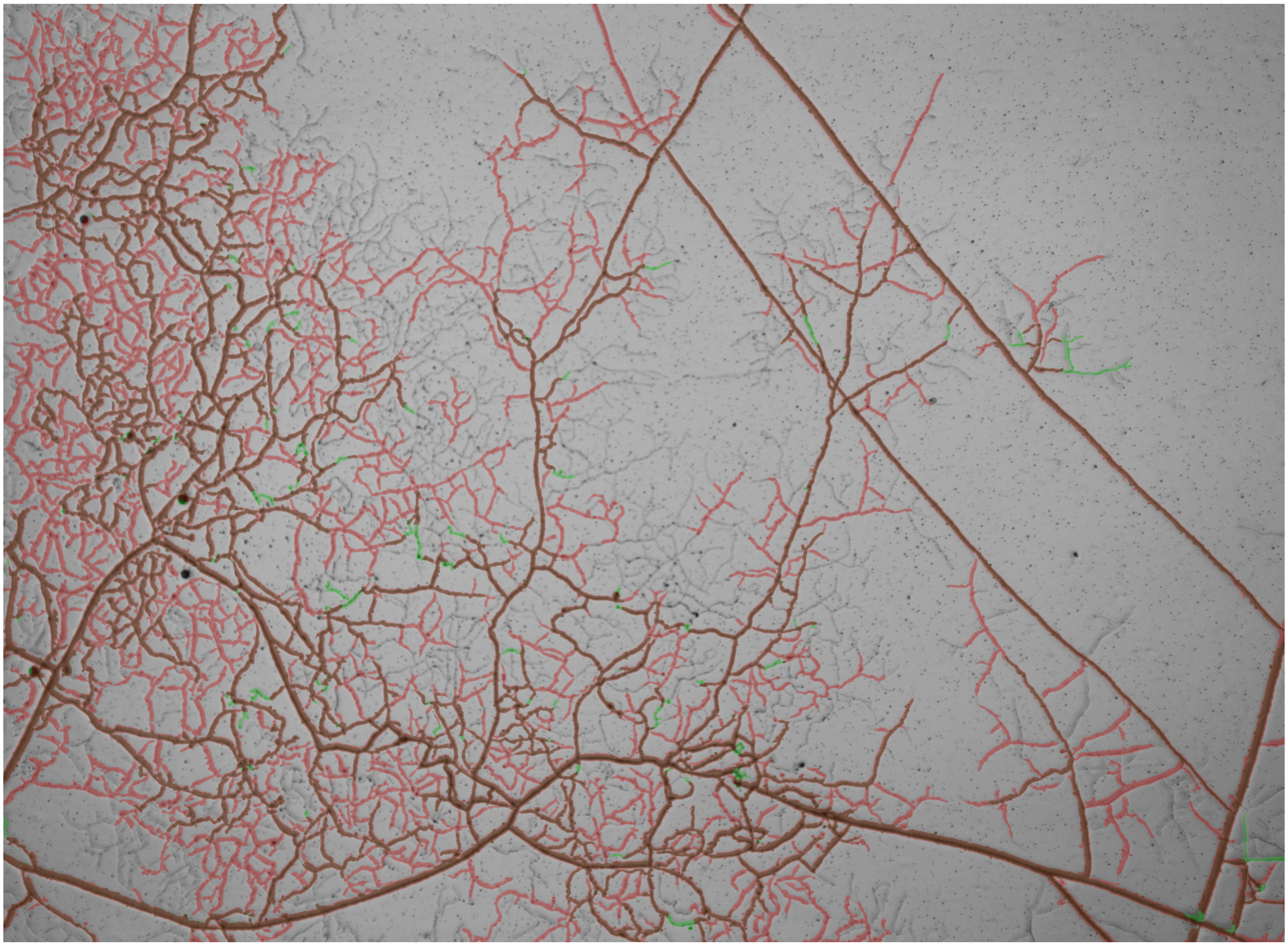

Fig. S9. U-Net segmentation captures many more hyphae in dense networks. The output of U-Net is shown in red, the classic segmentation method is in green, and brown regions are where both colors overlap.

7. SI References

References

1. L Oyarte Galvez, et al., A travelling-wave strategy for plant–fungal trade. *Nature* **639**, 172–180 (2025) Publisher: Nature Publishing Group.
2. J Murphy, JP Riley, A modified single solution method for the determination of phosphate in natural waters. *Anal. Chimica Acta* **27**, 31–36 (1962).
3. H Etesami, BR Jeong, BR Glick, Contribution of Arbuscular Mycorrhizal Fungi, Phosphate–Solubilizing Bacteria, and Silicon to P Uptake by Plant. *Front. Plant Sci.* **12** (2021).
4. O Ronneberger, P Fischer, T Brox, U-Net: Convolutional Networks for Biomedical Image Segmentation in *Medical Image Computing and Computer-Assisted Intervention – MICCAI 2015.* (Springer, Cham), pp. 234–241 (2015) ISSN: 1611-3349.
5. JSB IV, P Pessoa, M Tavakoli, S Presse, Perspectives: Comparison of Deep Learning Segmentation Models on Biophysical and Biomedical Data (2025) arXiv:2408.07786 [eess].
6. N Srivastava, G Hinton, A Krizhevsky, I Sutskever, R Salakhutdinov, Dropout: a simple way to prevent neural networks from overfitting. *J. Mach. Learn. Res.* **15**, 1929–1958 (2014).
7. I Sutskever, J Martens, G Dahl, G Hinton, On the importance of initialization and momentum in deep learning in *Proceedings of the 30th International Conference on Machine Learning.* (PMLR), pp. 1139–1147 (2013) ISSN: 1938-7228.
8. LR Bakken, RA Olsen, Buoyant densities and dry-matter contents of microorganisms: conversion of a measured biovolume into biomass. *Appl. Environ. Microbiol.* **45**, 1188–1195 (1983).
9. YM Bar-On, R Phillips, R Milo, The biomass distribution on Earth. *Proc. Natl. Acad. Sci.* **115**, 6506–6511 (2018) Publisher: Proceedings of the National Academy of Sciences.
10. I Jakobsen, L Rosendahl, Carbon flow into soil and external hyphae from roots of mycorrhizal cucumber plants. *New Phytol.* **115**, 77–83 (1990).
11. JJ Rautio, BA Smit, M Wiebe, M Penttilä, M Saloheimo, Transcriptional monitoring of steady state and effects of anaerobic phases in chemostat cultures of the filamentous fungus Trichoderma reesei. *BMC Genomics* **7**, 247 (2006).
12. AJ Saldanha, MJ Brauer, D Botstein, Nutritional Homeostasis in Batch and Steady-State Culture of Yeast. *Mol. Biol. Cell* **15**, 4089–4104 (2004).
13. A Schnepf, T Roose, Modelling the contribution of arbuscular mycorrhizal fungi to plant phosphate uptake. *New Phytol.* **171**, 669–682 (2006) _eprint: https://nph.onlinelibrary.wiley.com/doi/pdf/10.1111/j.1469-8137.2006.01771.x.
14. W Davison, *Diffusive Gradients in Thin-Films for Environmental Measurements.* (Cambridge University Press), (2016) Google-Books-ID: BFvWDAAAQBAJ.
15. SD Harris, Branching of fungal hyphae: regulation, mechanisms and comparison with other branching systems. *Mycologia* **100**, 823–832 (2008).
16. RL Bieleski, Phosphate Pools, Phosphate Transport, and Phosphate Availability. *Annu. Rev. Plant Physiol.* **24**, 225–252 (1973).
17. PR Darrah, S Staunton, A mathematical model of root uptake of cations incorporating root turnover, distribution within the plant, and recycling of absorbed species. *Eur. J. Soil Sci.* **51**, 643–653 (2000) _eprint: https://onlinelibrary.wiley.com/doi/pdf/10.1046/j.1365-2389.2000.00331.x.
18. L Chevalier, F Klingelschmitt, L Mousseron, N Minc, Mechanical strategies supporting growth and size diversity in Filamentous Fungi. *Mol. Biol. Cell* **35**, br17 (2024) Publisher: American Society for Cell Biology (mboc).
19. B Bago, C Azcón-Aguilar, A Goulet, Y Piché, Branched absorbing structures (BAS): a feature of the extraradical mycelium of symbiotic arbuscular mycorrhizal fungi. *New Phytol.* **139**, 375–388 (1998) _eprint: https://nph.onlinelibrary.wiley.com/doi/pdf/10.1046/j.1469-8137.1998.00199.x.
20. B Bago, et al., Translocation and Utilization of Fungal Storage Lipid in the Arbuscular Mycorrhizal Symbiosis. *Plant Physiol.* **128**, 108–124 (2002).
21. A Hagenbo, et al., Carbon use efficiency of mycorrhizal fungal mycelium increases during the growing season but decreases with forest age across a Pinus sylvestris chronosequence. *J. Ecol.* **107**, 2808–2822 (2019) _eprint: https://onlinelibrary.wiley.com/doi/pdf/10.1111/1365-2745.13209.
22. LLM Heaton, NS Jones, MD Fricker, Energetic Constraints on Fungal Growth. *The Am. Nat.* **187**, E27–E40 (2015) Publisher: The University of Chicago Press.
23. H Kameoka, T Maeda, N Okuma, M Kawaguchi, Structure-Specific Regulation of Nutrient Transport and Metabolism in Arbuscular Mycorrhizal Fungi. *Plant Cell Physiol.* **60**, 2272–2281 (2019).
24. S Manzoni, P Taylor, A Richter, A Porporato, GI Ågren, Environmental and stoichiometric controls on microbial carbon-use efficiency in soils. *New Phytol.* **196**, 79–91 (2012) _eprint: https://nph.onlinelibrary.wiley.com/doi/pdf/10.1111/j.1469-8137.2012.04225.x.
25. AM Makarieva, et al., Mean mass-specific metabolic rates are strikingly similar across life's major domains: Evidence for life's metabolic optimum. *Proc. Natl. Acad. Sci.* **105**, 16994–16999 (2008) Publisher: Proceedings of the National Academy of Sciences.
26. J Krissansen-Totton, S Olson, DC Catling, Disequilibrium biosignatures over Earth history and implications for detecting exoplanet life. *Sci. Adv.* **4**, eaao5747 (2018).
